# A systematic evaluation of digital nutrition promotion websites and apps for supporting parents to influence children’s nutrition

**DOI:** 10.1186/s12966-020-0915-1

**Published:** 2020-02-10

**Authors:** Dorota Zarnowiecki, Chelsea E. Mauch, Georgia Middleton, Louisa Matwiejczyk, Wendy L. Watson, Jane Dibbs, Anita Dessaix, Rebecca K. Golley

**Affiliations:** 1 Flinders University, Nutrition and Dietetics, College of Nursing and Health Sciences, Sturt Road, Bedford Park, SA 5042 Australia; 2 Cancer Council New South Wales, 153 Dowling Street, Woolloomooloo, NSW 2011 Australia

**Keywords:** Child food intake, Parents, Nutrition, Lunchbox, Digital, mHealth, eHealth, Mobile applications, Website

## Abstract

**Background:**

Globally children’s diet quality is poor. Parents are primary gatekeepers to children’s food intake; however, reaching and engaging parents in nutrition promotion can be challenging. With growth in internet and smartphone use, digital platforms provide potential to disseminate information rapidly to many people. The objectives of this review were to conduct a comprehensive and systematic evaluation of nutrition promotion via websites and apps supporting parents to influence children’s nutrition, from three different perspectives: 1) current evidence base, 2) end user (parent) experience and 3) current commercial offerings.

**Methods:**

Three systematic reviews were undertaken of (1) studies evaluating the effectiveness for digital platforms for improving nutrition in children and parents, (2) studies conducting user-testing of digital tools with parents, (3) websites and apps providing lunch-provision information to parents. Searches were conducted in five databases for reviews one and two, and systematic search of Google and App Store for review three. Randomised controlled trials, cohort and cross-sectional and qualitative studies (study two only) were included if published in English, from 2013, with the intervention targeted at parents and at least 50% of intervention content focused on nutrition. Search results were double screened, with data extracted into standardised spreadsheets and quality appraisal of included search results.

**Results:**

Studies evaluating digital nutrition interventions targeting parents (*n* = 11) demonstrated effectiveness for improving nutrition outcomes, self-efficacy and knowledge. Six of the included randomised controlled trials reported digital interventions to be equal to, or better than comparison groups. User-testing studies (*n* = 9) identified that digital platforms should include both informative content and interactive features. Parents wanted evidence-based information from credible sources, practical tools, engaging content and connection with other users and health professionals. Websites targeting lunch provision (*n* = 15) were developed primarily by credible sources and included information-based content consistent with dietary guidelines and limited interactive features. Lunchbox apps (*n* = 6), developed mostly by commercial organisations, were more interactive but provided less credible information.

**Conclusions:**

Digital nutrition promotion interventions targeting parents can be effective for improving nutrition-related outcomes in children and parents. As demonstrated from the lunchbox context and user-testing with parents, they need to go beyond just providing information about positive dietary changes, to include the user-desired features supporting interactivity and personalisation.

## Background

Globally children’s diet quality is poor. Current diets are characterised by inadequate intakes of foods such as vegetables and whole grains, in combination with excess intakes of nutrient-poor food and drinks [1, 2]. Poor diet quality is inversely associated with risk factors for chronic disease such as excess weight gain [3] as well as poorer child development outcomes [4, 5]. Influencing the settings where children live, learn and play is needed to improve the quality of what children eat and drink to support optimal growth, health and development [6].

Homes are a natural setting for nutrition promotion with 60–70% of children’s food intake provided from within this setting [7, 8] (Price 2014, unpublished findings). Parents are the primary “gatekeepers” within homes, serving as role models, determining food availability and setting the family norms that shape children’s habits [9]. Nutrition promotion interventions where parents are the primary change agent are effective [6, 10]. However reaching and engaging care givers in ways that are meaningful to parents has been identified as a consistent barrier to intervention fidelity and effectiveness in child nutrition promotion interventions [10].

Digital health provides the opportunity to enhance the reach, engagement and intensity of supporting parents to improve children’s diet quality. With 97% of Australian households with children under 15 years of age now having internet access at home [11], digital health interventions can provide practical support, remotely, interactively, and in context. Digital nutrition promotion aligns with how health information access has shifted from pamphlets to be online, reaching consumers where they are looking for the information [12, 13]. Additionally, digital interventions are highly scalable and have the potential to reach a diverse population. The popularity of digital health interventions in both the general public and in published literature makes it an important platform for exploration of current effectiveness and end user experience.

Children spend over 200 days each year at school, consuming around 40% of their daily food intake in this setting [14, 15]. In many countries the food that children consume at school is provided as packed lunches from the home setting [16]. Increasingly, packed lunches are also becoming a more common food provision model in early childhood and education settings. Parent engagement is a critical component to support and enhance the range of nutrition promotion interventions to improve children’s diet quality within education settings [17]. Traditionally, strategies to engage parents through schools have been limited to reinforcing what occurs in school and have been of low intensity (i.e. school newsletters) [17]. However, evaluations of school-day food provision digital interventions are emerging [18, 19]. Hence, a review of digital platforms that are already available to support parent’s lunchbox food provision can provide a useful case study to better understand ways to effectively engage and support parents via digital platforms.

Bringing together different perspectives and evidence is needed to solve complex challenges such as engaging and supporting parents to improve the quality of the foods they provide to their children. Reviewing the literature to evaluate the effectiveness of interventions that have been scientifically tested is important. Drawing on end-user perspectives provides equally important insights to ensure interventions meet the needs of end-users. The end user experience is a strong predictor of intervention fidelity as well as research translation and implementation. Evaluation of currently available websites and mobile applications (apps) can support future research innovation by informing the translation of novel and emerging technologies into the research setting. Therefore, this review includes three perspectives: 1) the current evidence-base, 2) the end user (parent) perspective and 3) current commercial offerings to undertake a comprehensive and systematic evaluation of digital nutrition promotion websites and apps for supporting parents to influence children’s nutrition. The three review objectives were to review the evidence for effectiveness of digital tools targeting parents (objective one), understand what parents want from digital tools (objective two), and review commercially available digital tools supporting parents’ provision of school lunches to children (objective three).

## Methods

Three systematic reviews of the peer-reviewed literature (objectives one and two) and digital tools (websites and apps) targeting parents to improve children’s nutrition were undertaken in October to November 2018. The initial aim of these reviews was to understand the evidence for the use of digital platforms as health promotion tools supporting parents to provide children with a healthy lunchbox. However, pilot searches to develop the search strategy indicated that there was limited published literature evaluating effectiveness and user-testing of child lunchbox digital interventions targeting parents. Therefore, the inclusion criteria of objectives one and two were widened to include evidence of digital platforms supporting parents to influence children’s nutrition more broadly. However, broadened criteria were guided by relevance and ability to apply the findings to the lunchbox context. Given the volume and scope of nutrition information available for parents online, retaining the focused topic for objective three allowed for more comprehensive review and interrogation of search results. The Preferred Reporting Items for Systematic Reviews and Meta-Analyses (PRISMA) statement was adhered to when conducting all three reviews [20].

### Objectives one and two: digital tool effectiveness and parent perspective

Two systematic searches were undertaken to identify peer-reviewed literature evaluating the efficacy of websites and apps as health promotion tools for improving children’s nutrition (objective one) and user-testing of child nutrition apps and/or websites conducted with parents (objective two).

#### Search strategy

Search strategies were developed and tested in Medline (Ovid capturing PubMed) and translated for use in EMCARE (Ovid), PsychINFO (Ovid), Scopus and ProQuest databases. For both searches, key search terms were combined using the AND/OR operators for the population (‘parent’, ‘family’, ‘child’), intervention (‘website’, ‘mobile applications’, ‘smartphone’) and outcomes (‘health promotion’, ‘nutrition’, ‘obesity’). Search terms were mapped to database specific subject headings where available (Full search provided in Additional file 1). For the review of effectiveness, population search terms were removed from the final search strategy to ensure all relevant studies were identified. For the review of end user perspective, additional search terms to capture user testing outcomes (i.e. user testing, user feedback, functionality) were included. Search results were combined in EndNoteX9 and duplicates removed, then uploaded into Covidence systematic review software [21] for screening. Hand-searching of reference lists of included studies and relevant reviews identified in the search was undertaken to identify additional studies.

#### Inclusion and exclusion criteria

Randomised controlled trials, cohort and cross-sectional studies, qualitative studies (objective two only) published from 1 January 2013 – October 2018 were included if they were published in English and conducted in Australia, New Zealand, United Kingdom, United States of America (USA), Canada or Western Europe (including Scandinavia). Digital interventions (websites or apps) targeting parents or families were included where the nutrition component was at least 50% of content. Studies were excluded if the target population was < 1 years of age (i.e, content focused on breastfeeding, infant feeding practices), targeted a population with chronic health conditions (i.e. diabetes), smoking cessation or alcohol intake, and the intervention was delivered solely via other digital technologies such as text messages, telemedicine or wearable devices. The original search date range (2008–2018) was restricted to 2013 to increase relevance of findings for current patterns of technology use, due to a shift in technology use and increase in use and accessibility of the internet in homes from 2013 [11, 12]. Additionally, for inclusion in objective one, studies needed to report child and parent outcomes (excluded if no child outcomes reported, included if parent outcomes with at least one child outcome reported) evaluating the effectiveness of the intervention in terms of dietary intake, knowledge, attitudes or self-efficacy. To be included in objective two, studies needed to report on user testing conducted with parents of an app or website in relation to children’s nutrition, obesity, or general healthy eating advice for parents. Studies reporting only user-testing conducted with children or adolescents, or evaluating dietary measurement apps and eHealth records, were excluded.

#### Study selection and data extraction

Studies were screened by two independent reviewers in Covidence [21], firstly by title and abstract and then by full-text. Discrepancies were resolved by a third reviewer. Data was extracted by one reviewer and checked by a second reviewer. Standardised data extraction tables were utilised to extract study information (year, country, study design, digital tool, participants, measures), and results. Quantitative data were reported as mean scores or percentages, and where available, effect sizes and *p*-values. For objective two, qualitative data were reported as grouped themes and findings, as they were described by study authors.

#### Quality appraisal

Quality appraisal of studies in objective one was conducted using the ‘Quality Assessment Tool for Quantitative Studies’ developed by the Effective Public Health Practice Project (EPHPP) [22]. The EPHPP tool has been evaluated for content and initial construct validity, inter-rater reliability and test-retest reliability [23]. Studies were graded as weak, moderate or strong against six criteria; selection bias, study design, confounders, blinding, data collection methods, and withdrawals and drop-outs. Studies were assessed by two independent reviewers, with scoring discrepancies resolved through discussion by the reviewers. Studies included in objective two included qualitative and cross-sectional study design and therefore were not graded against these criteria.

### Objective three: commercially available digital tools to support parent school lunch provision

#### Search strategy

Searches for both apps and websites were modelled on prior research [24–26]. Website searches were conducted in a Chrome browser set to ‘incognito’ mode. Search terms were determined using Google trends and pilot searches. Google Advanced settings were used to limit searches to English websites and combine search terms using ‘all of these words’ (lunch, lunch box or school lunch box) and ‘any of these words’ (ideas, tips, health(y), school, planner or planning tool). Four searches combining key search terms were repeated in each of the regions of interest, as below, resulting in a total of 20 searches. Searches were conducted in the Australia region, and then replicated in New Zealand, United Kingdom, Canada and the USA regions. The first 10 pages of each search (100 results) were screened. Additional hand searching of government and non-government websites was conducted to ensure all relevant websites were identified (nil further identified).

App searches were conducted in the AppStore on an Apple iPad Mini Version 4 (Model A1550). Other commercial app stores (i.e. GooglePlay) were not searched as prior work found that few apps were developed for other platforms exclusively [26]. Search terms were similar to those for websites, but included some broader terms (e.g. nutrition, healthy eating, family meals) due to limited search capability within the AppStore, which did not enable more than 2–3 search terms to be combined. Eighteen searches were conducted, with the first 50 results screened (except where the term returned less than 50 results).

#### Website and app selection

Apps and websites were first screened by title and description, followed by full screening of the digital platform against inclusion/exclusion criteria. Websites and apps were included where they supported parents (with or without their children) in packing a healthy lunchbox, or providing, promoting or planning healthy meals and snacks for the family. Included websites were either dedicated to lunch provision or contained a section with more than one page of lunch-provision information, with links, resources or an interactive component. Apps included those that incorporated a form of meal planner, shopping list or other household function where they directly related to the provision of lunch or lunchbox items. Recipe platforms (including blogs, new articles, magazines) with no other functionality supporting food provision, infant-feeding, children’s games, weight-loss focussed or platforms limited to general healthy eating advice were excluded. School canteen ordering apps with no nutrition educational content were also excluded.

Apps requiring a one-off payment to download were purchased, while freemium apps (those with further content available with payment) were assessed in the freemium state as upgrades did not alter functionality. Search results were entered into a purpose designed spreadsheet, and at each stage of screening a 10% sample was checked against inclusion/exclusion criteria by a second reviewer.

#### Data extraction

Apps and websites were used for at least 10 min prior to data extraction taking place. Website/app data including developer/author name, affiliation, year developed, purpose, target audience, key messages and features, functionality and technical features were extracted**.** Credibility of content was judged on the basis of information consistent with or reference to national dietary guidelines, or development of content by credentialed experts. Data extraction for all included apps and websites was checked for accuracy by a second reviewer.

#### App and website quality assessment

The Mobile App Rating Scale (MARS) was used to assess app quality [27]. The scale has been tested for reliability and includes domains measuring app aesthetics, functionality, information and engagement qualities [27]. The scale was then modified for the assessment of websites, with question wording modified for relevance to websites, and three items removed. MARS items were rated from 1 to 5, with five indicating the highest quality or best performing websites and apps. Two reviewers independently rated websites and apps, discussing discrepancies until consensus was reached. Mean domain scores and an overall MARS score (mean of all four domains) were calculated.

## Results

**Fig. 1:**
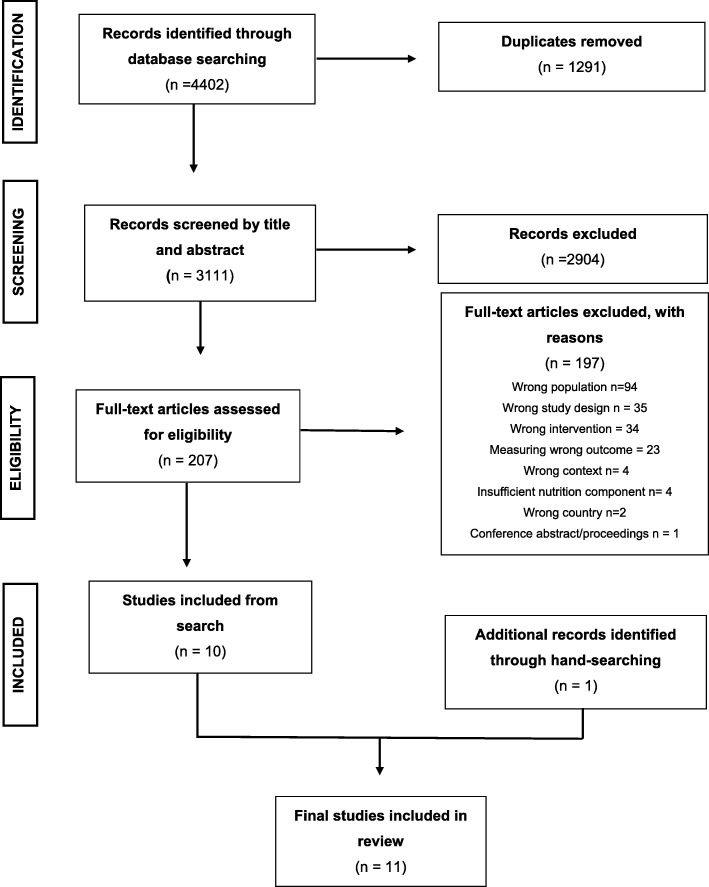
PRISMA Flow chart for article selection for objective one

**Fig. 2:**
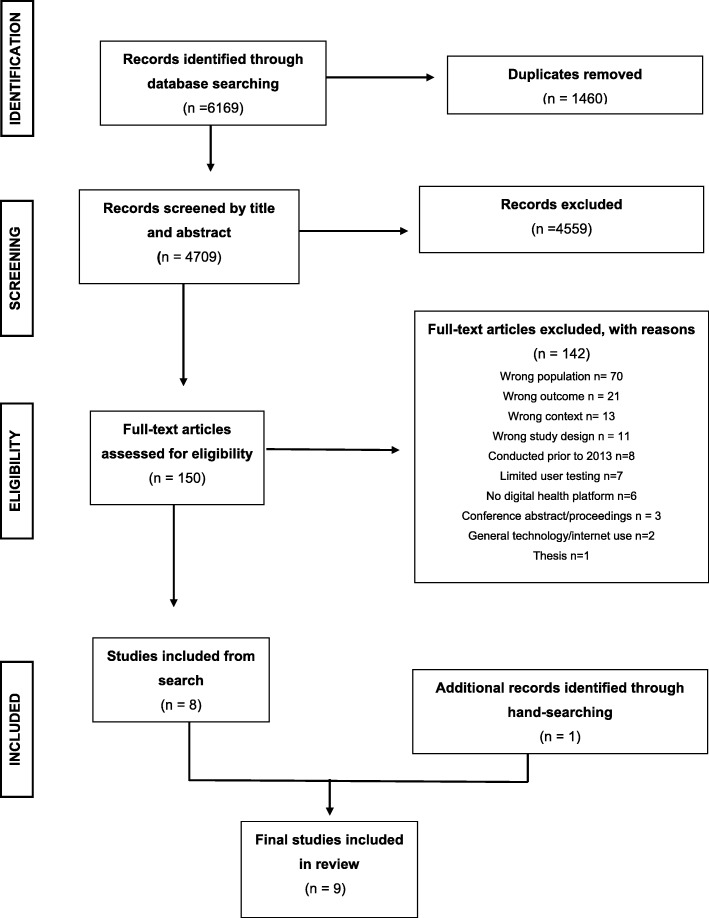
PRISMA Flow chart of article selection for objective two

**Fig. 3:**
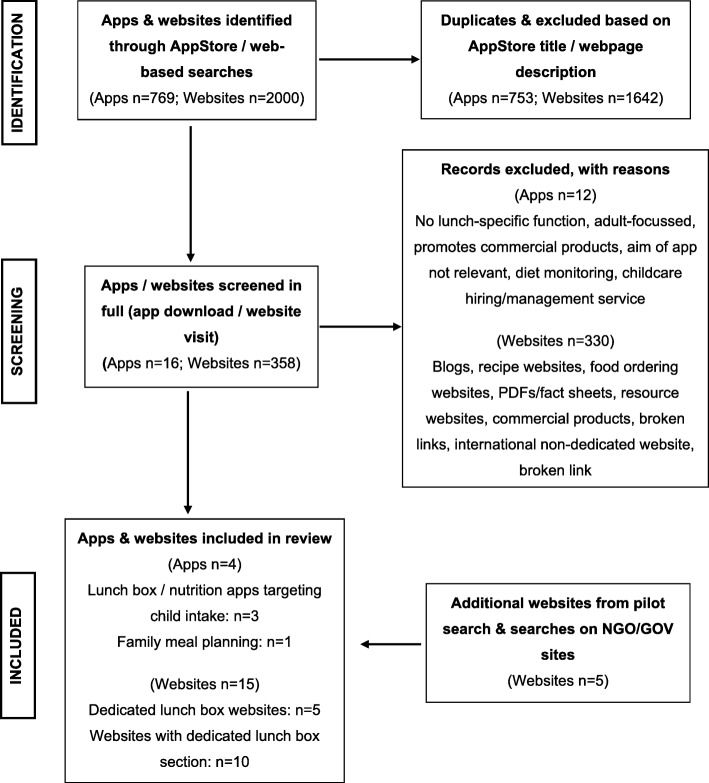
PRISMA Flow chart for website and app selection for objective three

Searches identified 4402 studies for objective one and 6169 studies for objective two (see Figs. 1 and 2 for PRISMA flow charts). Ten studies met inclusion criteria for objective one, and eight studies were identified for objective two. Two additional studies were identified through hand searching for objective one and objective two, bringing the final numbers to 11 studies for objective one and nine studies for objective two. Two thousand websites and 769 apps were screened, with 358 (18%) and 16 (2%) reviewed in their entirety for inclusion/exclusion (Fig. 3). Fifteen websites and four apps met inclusion criteria, representing less than 1% of those screened.

### Objective one – what is the effectiveness of digital nutrition promotion tools targeting parents?

#### Study characteristics

Eleven papers reporting on eight studies evaluating the effectiveness of websites (*n* = 7) [28–36] and apps (*n* = 1) [37, 38] as health promotion tools targeting parents to improve children’s nutrition and/or prevent obesity were included (Table 1). Studies were conducted in the USA [28, 30, 32–34, 36], Sweden [37, 38], Australia [31], Switzerland [35] and Belgium [29]. Studies targeted parents of children across a range of developmental stages from young children aged 1–5 years [28] to early adolescence [36]. Of included studies, four were randomised controlled trials (RCT), comparing the digital intervention against a healthy eating pamphlet [37, 38], healthy eating information delivered online [32–34], additional information provided by short message service (SMS) or email [35], or an in-person group education [28]. One non-randomised quasi-experimental controlled trial compared against a waitlist control [29] and three studies were a pre/post study design [30, 31, 36]. Interventions included varied components with little consistency between interventions, including information or educational modules, assignments or quizzes, instructional videos, interactive games, tracking of behaviours, goal setting, tips and advice, recipes, newsletters or weekly emails, forums, access to health professionals and personalised feedback. Intervention length ranged from a once-off online lesson [28] to 8-weeks [35]. Reported contact time ranged from 20 min (once-off online lesson [28]), 22 sessions of short 2-min videos delivered over four weeks (44 min total; [29]) and up to 115 min contact time over four weeks [31].

**Table 1:** Characteristics and outcomes of studies evaluating efficacy of children’s nutrition digital health interventions (Objective 1)

	Digital intervention, duration	Study design, participants, timepoints	Outcomes	Quality rating	Dietary intake	BMI/weight	Knowledge	Self-efficacy / SCT	Attitudes/ beliefs
Delisle Nystrom 2018 ^1^ [37]; Sweden	I: MINISTOP app (HE/PA); 6-months; content delivered bi-weekly; contact time not reported C: HE/PA pamphlet	RCT; Parents 4yo children; BL (*n* = 315), 6-month (n = 315), 12-month (*n* = 263)	• Fat mass (FM) [measured] • FV, lollies, SSB intake [Food photos on smartphone] • Composite score - FM, PA, sedentary time, FV, lollies, SSB	Strong	0 B (SSB, FV, lolly, score)	0 B (FM) + I (FFM)			
Delisle Nystrom 2017 ^1^ [38]; Sweden				Strong	+ I (SSB) 0 (FV, lolly) + I (Score)	0 B (FM)			
Grimes 2018 [31]; Australia	I: DELISH Website (Salt Ed); 5-weeks; content delivered weekly; contact time ~ 23 mins per session C: Nil	Pre/post; Child (mean 9.2y) & parent; BL (*n* = 83), 5-weeks (n = 83)	• Salt knowledge, attitudes, self-efficacy [Online Qus] • Salt intake [24 h urine], behaviours [Online Qus]	Moderate	0 (Salt intake) + (Salt behav)		+	+	0
Knowlden 2018 ^2^ [33]; USA	I: EMPOWER website (SCT-based Obes prev program); 4 weeks; content delivered weekly; contact time ~ 30 mins per session C: Online healthy lifestyle info	RCT; Mothers 4–6 yo child; BL (*n* = 57); 4-weeks (*n* = 51); 8-weeks (*n* = 50); 1 year (*n* = 44); 2 years (*n* = 37)	• Mother’s self-efficacy [Online Qus] • Child intake: FV; sugar-free beverages [Online Qus]	Moderate	+ I (FV) + B (SFB)			+/− I (FV env only)	
Knowlden 2016 ^2^ [32]; USA				Moderate	+ I (FV) + B (SFB)			+/− I (FV env only)	
Knowlden 2015 ^2^ [34]; USA				Moderate	+ I (FV) + B (SFB)			+/− I	
Rangelov 2018 [35]; Switzerland	C: Website only (HE/PA) I1: Website + SMS I2: Website + email 8-weeks; content delivered weekly; contact time not reported	RCT; Children grades 1–2 & parents; BL: (*n* = 774); 8 weeks (*n* = 608)	• Child intake [7-day food diary]	Weak	+ C (F) + C (Sweets) + I1 (V) 0 (all other)				
De Lepeleere 2017 [29]; Belgium	I: Website (Health promoting videos); 4-weeks; content delivered weekly; contact time ~ 2 mins per video (22 videos) C: Waitlist control	Quasi-experimental CT; Parents primary school age child; BL (*n* = 207); 1-month (*n* = 135); 3-month (*n* = 128)	• Parent self-efficacy; Parenting practices [Qus] • Child intake: FV, water, SSB, snacks [FFQ]	Weak	0 (FV, water, SSB, snacks)			+/− I	+/− I (parent practices high SES only)
Au 2016 [28]; USA	I: Website (BF Ed); 1 online lesson; contact time ~ 15–20 min C: 1 x in-person group ed. session	RCT; Mothers 1-5yo child BL, 2-month, 4-month (*n* = 590)	• BF knowledge, attitudes, self-efficacy [Qus] • Child, parent BF intake [Qus]	Moderate	+ I (BF)		+ B	+ B	+ I
Wilson 2014 [36]; USA	I: Website (FV); once-off module; contact time predicted ~ 45–60 min C: Nil	Pre/post; Parents & adolescents(mean 13.3y); BL (*n* = 47); 1-week (*n* = 41)	• Parent & adolescent FV intake [Screening Qus]	Weak	+ (FV parent, adolescent)				
Delamater 2013 [30]; USA	I: FIT-4-Health Website as information source; contact time not reported C: Nil	Pre/post; Families with overweight children 8-12y; BL & 4 weeks (*n* = 24)	• Self-efficacy • BMI z-score [measured] • Food intake [Qus] • Healthy lifestyle score (Food intake, PA, sedentary)	Weak	+/0 (HE, high vs low users) + (Score)	+/0 (high vs low users)		+ (high users)	
TOTAL					11	3	2	7	3

^1,2^Studies reporting follow-up findings from the same intervention

Abbreviations: *A* app; *Behav* behaviours; *BF* breakfast; *BL* baseline; *BMI* body mass index; *CT* controlled trial; *Ed* education; *Env* environment; *F* fruit; *FFM* fat free mass; *FFQ* food frequency questionnaire; *FM* fat mass; *FV* fruit and vegetables; *HE* Healthy eating; *Info* information; *Mins* minutes; *Nil* not present; *Obes prev* obesity prevention; *PA* Physical activity; *QE* quasi-experimental; *Qus* questionnaire; *RCT* Randomised controlled trial; *Pre/Post* pre-test post-test; *SCT* social cognitive theory; *SES* socioeconomic status; *SFB* sugar free beverage; *SSB* sugar sweetened beverages; *USA* United States of America; *yo* years old

Abbreviations to denote results: B=Significant change in both groups, no difference between groups; I=Significant effect in intervention group; C=Significant effect in control group; + = positive intervention effect/improvement (hypothesised intervention effect); − = negative intervention effect / worsening, 0 = no effect; +/0 or +/− = mixed results

#### Study quality

One study (two included papers) [37, 38] was rated strong quality, two studies (five included papers) were rated as moderate quality [28, 31–34] and the remaining four studies were rated as weak [29, 30, 35, 36]. Poor reporting of study design contributed to lower quality scoring. In particular, information about the validity and reliability of instruments and reasons for withdrawal were poorly reported. In general, studies did not score highly against study blinding criteria. While participant blinding would not be feasible, only one study reported that assessors were blinded to participant allocation [32–34].

#### Dietary intake

Included studies examined the effect of digital interventions on fruit and vegetable intake (*n* = 8) [29, 32–38], sugar-sweetened beverages (*n* = 3) [29, 37, 38], sweets/lollies (*n* = 3) [35, 37, 38], breakfast intake (*n* = 1) [28], salt intake and behaviours (*n* = 1) [31], snacks (*n* = 1) [29] and overall diet quality (*n* = 1) [30]. Findings were mixed across studies, however all but one study [29] found a positive effect of the digital intervention on child nutrition across a range of dietary outcomes (Table 1). Improvements in dietary intake ranged from small to moderate effects (Additional file 2).

Most studies evaluated outcomes related to intake of healthy foods. Increases in fruit and vegetable intake were reported in five of eight studies [32–34, 36, 39]. Fruit and vegetable intake improved in intervention compared with control groups in two studies [32–35]. Knowlden found an increase of 1.84 cups of fruit and vegetables at one year, which was maintained at two year follow-up [32–34]. Knowlden compared their EMPOWER website with online healthy lifestyle information [32–34]. Rangelov compared a website as a stand-alone intervention (control group), against the website with additional information delivered via SMS or email, finding a small increase in frequency of fruit intake in the website only group and small increase in vegetable intake in website with SMS group [35]. A once-off online program found improvements in both parent and adolescent fruit and vegetable intake of 0.5–0.7 serves one-week after program completion [36]. Three papers reported no effect on fruit and vegetable intake [29, 37, 38]. Two studies evaluated measures of overall diet quality. Delamater [30] reported improvements in healthy eating behaviours in sub-group analyses that compared a small sample of high (*n* = 9) versus low users (*n* = 9) of the website. The MINISTOP app, compared with an healthy lifestyle pamphlet, improved a health behaviour score that included fruit, vegetable and sugar sweetened beverage intake at 6-months [38], however this was not maintained at 12 months [37]. Online lessons delivered via a website were shown to be more effective than a face-to-face group education for improving breakfast frequency and types of foods consumed in low income parents and children [28].

A small number of studies targeted changes in nutrient-poor outcomes. One of three studies found a positive effect of the digital intervention of reducing intake of sweetened beverages [29, 37, 38]. Delisle Nystrom found a small reduction of 12 ml/day (*p* = 0.49) of sweetened beverage intake compared with a small increase in the control group at 6-months [38], which was not maintained at 12-months [37]. One further study found a positive effect on reducing intake of sugar-free beverages, which was maintained at one and two years [32–34]. A 5-week salt education program delivered via a website found no effect on children’s salt intake, but found a decrease in use of salt at the table during meals [31].

#### Behavioural determinant outcomes

Six of seven website interventions evaluating self-efficacy found some positive effects on self-efficacy, of which five evaluated parents’ self-efficacy [28, 29, 32–34] and two evaluated children’s self-efficacy [30, 31]. Knowlden evaluated mother’s self-efficacy, home availability, emotional coping (i.e. managing child’s negative reactions) and outcome expectations for child intake of fruit, vegetables and sweetened beverages [34]. No effect on self-efficacy at any time points were found [32–34]. A positive effect was observed on mother’s self-reports of home availability and outcome expectations for fruit and vegetables in the intervention compared with control [34], which were only maintained for home availability at one and two years [32, 33]. Two pre-post studies found that children’s self-efficacy for reducing salt intake, healthy eating and weight management behaviours significantly improved following the website interventions [30, 31]. Studies evaluating nutrition knowledge [28, 31], attitudes [28, 31] and parenting feeding practices [29] found the website interventions to have significant positive effects on these outcomes, with the exception of Grimes whereby a salt education website improved salt knowledge and self-efficacy, but had no effect on attitudes to salt [31].

### Objective two – what do parents want from digital tools supporting children’s nutrition?

#### Study characteristics

Nine studies were identified reporting user-testing by parents of digital health tools targeting improvements in children’s nutrition. Five evaluated apps [18, 40–43], three evaluated websites [19, 39, 44] and one evaluated both an app and website [45] (Table 2; Additional file 3). Studies were conducted in the USA [41–44], Australia [18, 19, 45], Canada [40] and Switzerland [39]. Evaluations of preferences for content, features, technology, delivery mode, useability and barriers were conducted using focus groups [39–42, 44] and questionnaires [18, 19, 39, 41, 43, 45]. Given the small number of included studies and overall consistent findings across apps and websites, reporting of findings have been combined.

**Table 2:** Summary of user-preferred content, features and technology of digital platforms (Objective 2)

	Biediger-Friedman 2018 [41]	Luesse 2018 [44]	Reynolds 2018 [18]	Hull 2017 [43]	Wyse 2017 [19]	Avis 2016 [40]	Biediger-Friedman 2016 [42]	Burrows 2015 [45]	Rangelov 2015 [39]
Country and digital platform	USA, A	USA, W	AUS, A	USA, A	AUS, W	CAN, A	USA, A	AUS, WA	SWI, W
Study design	FG, Q	FG	Q	Q	Q	FG	FG	Q	FG, Q
Participants	*n* = 48 low income mothers	*n* = 16 low income parents	*n* = 196 primary school Principals	*n* = 63 mothers	*n* = 123 primary school Principals	*n* = 38 HP, parents, researcher	*n* = 64 low income mothers	*n* = 75 parents	FG n = 64 Q *n* = 759 Parent, child
CONTENT	✓	✓				✓		✓	✓
Specific and relevant for child and parents (i.e. themed, child-friendly food ideas), rather than general information (i.e. portions for different ages, recipes)	✓	✓				✓		✓	✓
Tailored/personalised content/feedback		✓				✓			
Trusted information, endorsed by University or Government organisation (voice of authority)	✓	✓						✓	
Information on multiple health behaviours (i.e. diet, physical activity, sedentary, BMI)	✓					✓			
Positive/affirming content, rather than negative content (i.e. disliked terminology about child obesity and weight management focus)						✓		✓	
Practical information leading to behaviour change (i.e. address barriers, motivates change, ways to improve behaviour and maintain changes)						✓			✓
Suitable for low-literacy (i.e. pictures, visual content)		✓							
Images / content reflecting cultural diversity						✓			
Content that can initiate conversation with paediatrician						✓			
Budget friendly information using everyday foods								✓	
FEATURES	✓	✓	✓	✓			✓	✓	✓
Features to involve the whole family (i.e. games area/ child activities, cooking with children, sections for parents)	✓						✓	✓	✓
Ability to post questions to health professionals (i.e. via a live chat interface, contact box, video chat) or regular contact with health professionals	✓						✓	✓	✓
Features facilitating community; Ability to connect/interact with other users including via a discussion forum, social media, Facebook chat (incl. Sharing information through social media, milestones, ideas, achievements, challenges)	✓		✓				✓	✓	✓
Engaging and interactive components (i.e. videos, games, quizzes, activities)	✓						✓	✓	✓
Practical tools and information (i.e. Shopping tools, barcode scanner, calculators snack gallery/ideas for healthy snacks, tips to buy/eat more FV, cooking demonstration videos, cooking techniques)	✓	✓		✓			✓		✓
Recipes (i.e. quick, cheap, child-friendly, healthy)		✓		✓					✓
Logging & tracking features for diet, exercise (Incl. tracking progress & awards for completion)	✓						✓		
Achievable and monitored goal setting, and with feedback		✓						✓	
Resources related to local area (i.e. local farmers markets)							✓		
FUNCTIONALITY	✓	✓		✓			✓	✓	✓
Mixed opinions about receiving communication / reminders / push notifications / messages, including via email or SMS. Disliked by some; preferred infrequent (i.e. < 1/week)		✓		✓				✓	✓
Customisable home page and personal user accounts							✓	✓	
Motivational prompts (challenges, pings, reminders, celebratory signals)	✓								
Enter goals via website or smartphone – i.e. flexible access via website or phone app								✓	
Library feature with search function							✓		
DELIVERY MODE	✓	✓						✓	✓
Website, email preferred								✓	✓
Mixed preference for info delivery outside of website (via text) and wanted print materials for children		✓							✓
Smartphone use, frequent app usage (few times per day or week); App is suitable	✓								
Access via website and app								✓	
Cross-modal – website, emails, text message, social media		✓							
Evening preferred time to receive content		✓							
Informal program with no scheduled sessions								✓	
USEABILITY, APPEAL & BARRIERS	✓		✓	✓	✓	✓		✓	
Self-explanatory, easy to use and helpful tools	✓			✓				✓	
Cost influential factor / low cost			✓					✓	
Clear instructions, descriptions and terms						✓			
Barriers: (lack of) internet access and set-up time (school canteen app), difficult to use					✓				

Abbreviations: *A* app; *AUS* Australia; *BMI* Body Mass Index; *CAN* Canada; *FG* Focus Group; *FV* fruit and vegetables; *HP* healthcare professionals; *Q* Questionnaire; *SWI* Switzerland; *USA* United States of America; *W* website; *WA* Website & app

#### Preferred content

Five studies evaluated preferences for content of digital health interventions [39–41, 44, 45]. Most prominently, participants noted that they wanted specific and relevant content, such as portion sizes for different ages, and relevant for all family members, especially the child (i.e. appropriate recipes) [39–41, 44, 45]. Parents disliked general or vague information, wanted to be able to access content that was tailored or personalised [40, 44], and desired practical information that supported behaviour change, for instance by addressing barriers [39, 40]. Three studies noted that parents wanted trustworthy, evidence-based information that was sourced from, or endorsed by, Universities or Government organisations [41, 44, 45]. Two studies also identified that parents wanted positive content, and that they disliked content and terminology focused solely on obesity and weight management, which elicited negative reactions such as fear, guilt or shame [40, 45].

#### Preferred features and functionality

Preferred features of digital tools were evaluated in seven studies [18, 39, 41–45]. Consistent with findings for preferred content, parents wanted features that could involve the whole family [39, 41, 42, 45] and wanted features to be both informative and practical [39, 41–44]. Favoured practical tools and information included shopping tools, budgeting, calculators, tip sheets, recipes and barcode scanners. Preferences for engaging and interactive features were reported, including videos (i.e. online cooking demonstrations), games and quizzes [39, 41, 42, 45], as well as logging and tracking features for diet and exercise with feedback [41, 42]. Features that enabled connection and/or interaction with other users and with health professionals were favoured across several studies [18, 39, 41, 42, 45]. Parents wanted to connect and interact with other users through discussion forums and social media to share information, ideas, achievements and challenges. The ability to interact with health professionals, to post questions, set goals and receive feedback on progress was also a desired feature [39, 41, 42, 45].

#### Functionality and delivery mode

Library features with search function, a customisable home page and personal user accounts were also sought after by parents [41–45]. Websites and apps were both found to be acceptable modes of intervention delivery, along with additional information and contact delivered via emails, text messages and social media [39, 41, 44, 45]. Use of push notifications, reminders or messaging (via text message or email mailing list) was evaluated, with somewhat mixed findings [39, 43–45]. Most users wanted to receive notifications or messages and were clear about not wanting to receive them too frequently, however there was no consistency about what frequency was acceptable. Some parents disliked notifications/emails and disabled push notifications on their apps, and another study found that information should be delivered no more than once per week [39]. One study found that participants wanted to receive motivational prompts, such as challenges, pings, reminders and celebratory signals [41].

#### Useability, appeal and barriers

Parents wanted digital tools to be self-explanatory, useful and easy to use [40, 41, 43, 45]. Two studies found that cost was an influential factor and tools should be low or no cost [18, 45]. One study evaluating school Principals’ perceptions of barriers for schools and parents’ use of apps, noting lack of internet, difficulty of use and set-up time (for the school) to be key barriers to use [19].

### Objective three - what digital tools (websites and apps) supporting parent provision of school lunches to children are currently available?

#### Website and App Characteristics

Of the 19 included websites (*n* = 15) [46–60] and apps (*n* = 4) [61–64], 12 websites (80%) were Australian [46, 47, 51–60], while all remaining websites and apps were developed overseas [49, 50, 61–65] (Table 3, Fig. 3 PRISMA). One third (*n* = 5) of websites were focussed on children’s lunchboxes [46–50], while 10 were broader websites with lunchbox sections [51–60]. Two of the four apps were focussed on children’s lunchboxes [62, 63], with the two remaining apps providing lunchbox information within an app about family meals [61, 64].

**Table 3:** Summary of website and app content and characteristics (Objective 3)

Name, country	Affiliation / developer	FEATURES									Mobile App Rating Scale (0–5)				
		Interactive lunch box builder	Person-alisation	Lunch box recipes/ ideas	Nutrition info (lunch box or general)	Games and/or quizzes	Video or audio content	Social commu-nity	Links to further info	School / canteen relevant info	Engagement	Functionality	Aesthetics	Information quality	Overall score
WEBSITES															
Healthy Lunch Box[1] [46] Australia	Cancer Council New South Wales	✓		✓	✓			✓	✓		3.8	4.7	4.0	4.2	4.2
Go For Your Life - Weigh up your lunch [47] Australia	Dept of Health Victoria	✓			✓	✓			✓		4.5	3.7	4.3	3.3	4.0
Healthier Lunches for Children [48] United Kingdom	London Borough of Islington				✓	✓		✓	✓	✓	3.0	4.0	2.3	3.8	3.3
The Zero Waste and Healthy Lunchbox [49] Canada	NWRSC Solid Waste Service				✓		✓			✓	2.8	4.0	3.3	3.0	3.3
Healthy Lunch Box[2] [50] N/A	Individual developer			✓	✓	✓		✓		✓	2.3	2.7	3.0	1.3	2.3
Make Healthy Normal: Healthy School Lunch Box [51] Australia	Dept of Health New South Wales	✓	✓						✓		4.0	4.0	4.0	3.2	3.8
Healthy Kids Association: Packing a healthy lunchbox [52] Australia	Healthy Kids Association			✓	✓			✓	✓		3.3	4.0	4.0	3.7	3.7
Nutrition Australia: Healthy lunchbox week [53] Australia	Nutrition Australia			✓	✓			✓	✓		3.5	3.7	3.3	4.0	3.6
Healthy Kids NSW: Lunch Box Ideas [54] Australia	Ministry of Health New South Wales & others			✓	✓				✓		3.0	3.7	3.7	3.7	3.5
Nestle Healthy Active Kids: What makes a balanced lunchbox? [55] Australia	Australian Institute of Sport & Nestle			✓	✓				✓	✓	3.5	3.7	3.7	3.3	3.5
Healthy Eating Advisory Service: Healthy lunchboxes [56] Australia	Healthy Eating Advisory Service & Nutrition Aust			✓	✓			✓	✓		3.0	4.0	3.0	3.7	3.4
QLD Education: A healthy start to school tool kit [57] Australia	Dept of Education Queensland			✓	✓			✓	✓	✓	2.3	4.0	3.0	3.8	3.3
WA School Canteens: Packing healthy lunchboxes [58] Australia	School Canteen Assoc Inc. Western Australia			✓	✓			✓	✓	✓	2.3	4.0	2.7	3.5	3.1
TAS Health: Healthy kids lunchboxes [59] Australia	Dept of Health & Human Services Tasmania			✓	✓			✓	✓		3.0	2.3	3.3	3.7	3.1
SA Health: Healthy lunchboxes [60] Australia	SA Health			✓	✓			✓	✓		2.5	3.7	2.3	3.6	3.0
APPS															
The Ultimate Mix-and-Match School Lunchbox [63] United States of America	Trellisys.net	✓	✓	✓	✓			✓			3.2	3.8	3.3	3.2	3.4
LaLa Lunchbox [62] USA	Lala Lunchbox, LLC	✓	✓	✓				✓			3.4	3.8	3.0	3.0	3.3
Kids Foods [61] Vietnam	Individual developer		✓	✓	✓						2.4	3.8	2.3	2.8	2.8
Change4Life Smart Recipes [64] United Kingdom	Public Health England		✓	✓	✓		✓	✓			3.0	3.5	2.7	4.0	3.3

#### Websites

Websites were mostly developed by, or in partnership with, government departments (national n = 1, state *n* = 8 and local *n* = 2) [47–49, 51, 54–60] and/or non-government organisation (NGO) (*n* = 6) [46, 52–54, 56, 58]. Websites were targeted at parents (*n* = 14) [46, 47, 49–60], children (*n* = 3) [46, 47, 51] and/or school policy/curriculum (*n* = 7) [48–50, 54, 55, 57, 58]. Information contained in all but one of the websites was consistent with their respective national guidelines [46–49, 51–60]. Most (*n* = 13) were information heavy, primarily in the form of text and images, either directly within the website or as downloadable PDF documents [46, 48–50, 52–60]. Most (*n* = 11) included key messages regarding the inclusion of food from the five food groups [46, 48, 50, 51, 53–56, 58–60], with other key messages addressing limiting nutrient-poor foods, and/or including water in the lunchbox (Described in more detail in Additional file 4). Two websites incorporated interactive features focussing on the practical aspects of building a healthy lunchbox [46, 51], and one included activity designed to teach parents/children about the healthfulness of lunchbox choices [47]. A further three websites included interactive games targeting children [47, 48, 50]. Website quality was rated a mean of 3.4 out of five, using a modified version of the MARS. Functionality (describing how the website functioned and ease of use) was the domain with the highest overall rating of 3.7, and engagement (describing website interactivity and entertainment) scored lowest at 3.1.

#### Apps

Only one app was developed by a government organisation, and this was the only free app of those included [64]. Two of the remaining three apps had optional paid upgrades (i.e. were ‘freemium’) [62, 63] and one had a once-off fee to purchase [61]. The two apps targeting lunchboxes specifically were usable by both parent and child, with one specifically encouraging collaborative choices (i.e. parent-controlled lunchbox options, with children able to choose from these options) [62, 63]. The remaining two apps provided recipes and nutrition information intended for parents [61, 64]. Foods from the five food groups were the focus of apps, with fruit and vegetables prominent, however the commercially developed apps showed no evidence of engagement with health professionals in the development of content [61–63]. All apps allowed some level of personalisation and included push notifications as reminders to plan lunches or purchase groceries. The two lunchbox focussed apps were interactive but contained little information other than lunch ideas or recipes [62, 63], whereas those that provided more content were less interactive [61, 64]. App quality was similar to that of websites, producing a mean MARS of 3.3, with the functionality domain the highest at 3.8 and engagement the lowest at 3.0.

## Discussion

Unique to this study was that three systematic reviews were undertaken considering the perspectives of the researcher, end-user and developer to evaluate the effectiveness, user applicability and utilisation of nutrition promotion web-based programs and apps for supporting parents to influence children’s nutrition. This review provided evidence that website and app-based interventions can be effective for improving parent’s and children’s dietary intake, nutrition knowledge and self-efficacy. However, the small number of studies identified, and wide range of outcomes evaluated limited conclusions which could be drawn. User engagement, which is an important determinant of intervention effectiveness [26, 66], was seen to be a challenge in evaluated studies. Similarly, many websites and apps identified in objective three scored poorly for engagement. Accordingly, user-testing showed a preference for credible information, on a platform that was engaging, personalised and interactive, and yet there was a tendency for the evidence-based websites to favour passive content and for interactive apps to lack evidence-based content. Despite the ubiquitous availability of smartphone apps and web-based programs there were few dedicated digital tools available at scale supporting parents to provide nutritious lunch foods and drinks.

Digital interventions appear to be a promising avenue for improving children’s nutrition and are an intervention approach that is aligned with shifts in society and trends for how health information is accessed [12, 13]. Parental use of web-based programs and smartphone apps is high, and parents feel confident using the internet and apps on smartphones, reflecting societal trends in digital technology use [12, 41, 67]. This review suggests that nutrition promotion websites and apps can achieve small to moderate changes in fruits, vegetables and nutrient-poor foods and drinks [31–34, 36, 38, 39], albeit with more studies needed to further substantiate their effectiveness. A recent Australian study, which included lunchbox messages provided to parents via a school communication app as part of a multi-component school-based intervention, found small improvements in the energy intake from recommended foods in the lunchbox [68]. Promisingly, 89% of parents downloaded the app, 71% recalled receiving health promotion messages and most found the messages acceptable and helpful, indicating feasibility and acceptability [68]. Digital interventions delivered to parents in other contexts support the use of apps and websites as an effective intervention mode for improving health behaviours. Digital tools promoting healthy infant feeding practices with mothers have shown promising results [69, 70], as have studies evaluating the effects of app-based nutrition promotion interventions during pregnancy although improvements compared to controls were not statistically significant [71]. Improvements equal to or slightly better than comparison groups suggest that digital nutrition promotion interventions are at least similar to conventional nutrition intervention delivery modes such as face-to-face programs and pamphlets [28, 38]. This is consistent with evaluations of other apps and web-based interventions which report that digital tools have similar effect to paper-based or face-to-face comparison groups [72] and offer no additional benefits if delivered with non-digital interventions [73]. Also consistent with other systematic reviews of digital health promotion interventions is evidence of short-term changes in health behaviours, but the long-term efficacy is yet to be determined [72, 74]. It is unclear what dosage and intervention length are needed to achieve behaviour change, but findings of this review suggest that interventions with low dosage and contact time such a once-off online lessons [28, 36] or short contacts (i.e. 2-min) over multiple occasions [35] can be effective for improving dietary intake.

A key constraint identified as limiting the impact of digital nutrition promotion interventions was a lack of initial and sustained engagement. Poor or limited engagement reduces effectiveness even if the digital intervention and behavioural strategies are well developed [70]. Parent’s engagement with digital tools is critical if parents are to be exposed to the behaviour change strategies underpinning these interventions [75]. Despite high initial visitation and engagement, many digital health interventions are limited by poor repeat visitation, ongoing adherence and reduced time spent engaged with the digital tool over the duration of the intervention [76, 77]. In an evaluation of a web-based intervention for families with overweight children, few program effects were noted but sub-group analysis showed that high users had significant improvements compared to low-users [30] suggesting that sustained engagement is paramount. Strategies that therefore improve user engagement and enable ongoing and repeated exposure will support intervention effects [75]. The strategy used to reach intended users will also influence engagement with digital nutrition promotion interventions. Social media approaches (predominately Facebook, Instagram and targeted website advertisements) have a wide reach and are low cost but are less effective at reaching target users [78, 79]. In comparison, traditional methods such as word-of-mouth and paper-based marketing reach fewer people but result in better access to target participants [78, 80]. Leveraging both social media and traditional means of marketing may achieve reach and awareness, as well as targeting and engaging with more intended users.

Understanding the unique needs of the intended users and what they want is key to designing digital tools that facilitate sustained engagement [81]. User-testing and process evaluation identified that parents wanted digital platforms that provided evidence-based, credible content from trusted sources, as well engaging, personalised and practical information. Moreover, features that enabled interaction with health professionals and other users were important to parents. These findings are consistent with user-testing for other health-related apps [69, 70]. Overall, websites and apps in use currently as health promotion tools targeting parents reviewed here did not meet the combination of desired features and content identified in user-testing. Websites provided considerable information content and few interactive features, with one notable exception which scored well for both engagement and information quality [46]. Apps tended to provide more interactive features, links with social media and personalisation, but less information content from credible sources. Thus, there is a need to optimise the design of nutrition promotion delivered to parents via digital tools to meet to desired combination of features, which may improve engagement.

A particular gap in the market appears to be a lack of apps that provide credible evidence-based nutrition information, in combination with interactive and collaborative features. An app could allow for greater personalisation of content and provide ‘real time’ reminders to perform target behaviours (such as nightly reminders to plan lunchboxes for the next day), both of which are may improve the engagement quality of such a program. Despite a paucity of studies examining the long-term efficacy of digital nutrition promotion interventions, addressing issues of reach, awareness, engagement and end-user co-designed products are more pressing priorities. Co-designing apps with end-users, developers and nutrition experts and adding credible information, interactivity and features to increase sustained, ongoing engagement would strengthen the potential of nutrition promotion apps to improve children’s nutrition [82]. Apps are of interest to both researchers and commercial developers and by collectively modifying existing tools further gains could be achieved. Evaluation research is also needed to determine a sufficient level of engagement to achieve behavioural change, as engagement appears to be the factor constraining the effectiveness of digital nutrition promotion interventions [83]. When the product meets the perceived needs of the end-user and can achieve sustained engagement, long-term efficacy can be measured.

Governments have a role to play in disseminating evidence-based health information in a form that translates that evidence into practical information, with the ability to reach and support the most disadvantaged [84]. This review of user-testing studies (objective 2) demonstrated that parents want information to be provided by trusted voices of authority such as Government organisations or Universities. As we also showed, not-for-profit organisations can also play a role although they often have limited access to funding sources [85]. Information delivered by a credible source and free of commercial conflict of interest is essential to maintain consumer trust in that information. The role of the food industry in public health initiatives has been debated given the food industry’s promotion activities and commercially driven goals [86]. This is a complex issue that requires consideration that falls outside the scope of this review. However, beyond the food industry, public-private collaboration, in particular with digital industries, is increasingly being recognised as an important part of the co-design process of digital health interventions and may take forms from advisory roles to partnerships [82, 87]. Industry partners and developers generally have more direct access to the target population that health interventions are seeking to reach and the knowledge, skills, financial backing and technology to enable rapid intervention development and dissemination [82]. For successful private-public collaboration, it is critical potential conflicts of interests are considered and that the objectives and outcomes are shared by all parties.

A key strength of this review was the evaluation of both grey and peer-reviewed literature, providing unique evidence regarding the use and effectiveness of web-based programs and apps in nutrition promotion. This review used systematic search strategies and established methodologies [20], utilising dual reviewers to ensure the accuracy of data extraction and assessment. A limitation of the systematic review component of this study was the heterogeneity in study design (including comparison to similar platforms, rather than traditional interventions such as face-to-face) and outcome measures. Further limitations included the small number of eligible publications and apps, discrepancies in inclusion/exclusion criteria in order to ensure a manageable sample of websites and in the extraction and assessment of content across two quite different platforms. Furthermore, the adaptation of the existing MARS tool [27] for use in websites means that app and web-based program quality ratings are not directly comparable. Only studies published in English from selected countries with comparable food supply were included, which may impact on the generalisability of the findings. Interestingly, there was considerable similarity in the design of digital interventions across included studies despite variation in child age and developmental stage, ethnicity and socioeconomic status of participants, suggesting acceptability and feasibility of digital health promotion targeting parents across a range of population groups.

## Conclusions

The use of web-based programs and smartphone apps will continue to proliferate as health information services used by the public [88]. Digital nutrition promotion interventions provide an opportunity to address the public health issue of improving children’s nutrition, with results to-date suggesting that these interventions can be effective for improving nutrition-related outcomes. Web-based programs and apps are relatively low cost with the potential for broad reach, however sustained engagement is a key factor constraining effectiveness. To address this, digital nutrition promotion aimed at parents needs to go beyond just providing information about positive dietary changes [89, 90], to include the user-desired combination of credentialled information, interactivity, personalisation and tailored feedback. There are opportunities for further development and evaluation, particularly within the app market, to establish long-term efficacy. Children’s school lunchboxes present a discreet behavioural target that could be addressed using digital technologies, however future solutions should be co-designed with end-users, developers and nutrition experts to promote maximum engagement and improve efficacy.

## Supplementary information

**Additional file 1.** Full example search strategy for objectives one, two, and three. Full search strategy for Ovid MEDLINE database for objective one and objective two, and full search strategy for websites and apps for objective three

**Additional file 2.** Data extraction table for studies evaluating effectiveness of digital health platforms for improving nutrition outcomes. Table of data extracted from included studies regarding the effectiveness of the digital health platforms

**Additional file 3.** Characteristics and outcomes of user-testing studies (objective two). Table of study characteristics and outcomes of user-testing from included studies

**Additional file 4.** 4.1 - Summary of included websites (objective three); and 4.2 - Summary of included apps (objective three). Tables of characteristics and summary of websites and apps included in review

## Data Availability

Not applicable.

## Abbreviations

Apps: Applications
EPHPP: Effective Public Health Practice Project
MARS: Mobile App Rating Scale
RCT: Randomised controlled trials
SMS: Short message service
USA: United States of America
