# Supplementary material for: A systematic evaluation of digital nutrition promotion websites and apps for supporting parents to influence children’s nutrition
Source: Int J Behav Nutr Phys Act. 2020 Feb 10;17:17. doi: 10.1186/s12966-020-0915-1 (PMC7011240; doi:10.1186/s12966-020-0915-1)
Supplement: Supplementary file 1 — Additional file 1. Full example search strategy for objectives one, two, and three. Full search strategy for Ovid MEDLINE database for objective one and objective two, and full search strategy for websites and apps for objective three [file 12966_2020_915_MOESM1_ESM.docx]

Additional File 1 – Full example search strategy for objectives one, two, and three

Objective one: Database(s): Ovid MEDLINE(R) and Epub Ahead of Print, In-Process & Other Non-Indexed Citations, Daily and Versions(R) 1946 to October 22, 2018
Search Strategy:

| **#** | **Searches** | **Results** |
| --- | --- | --- |
| 1 | Mobile applications/ or *Webcasts as topic/ or *social networking/ or *smartphone/ or *text messaging/ or video games/ | 11811 |
| 2 | (webcast* or podcast* or mobile application* or smartphone* or iphone or android or text messag* or app or apps or virtual reality or ipad* or tablet comput* or tablet device* or instant messag* or SMS* or whatsapp or Youtube* or video gam*).tw,kf. | 51803 |
| 3 | (e-health* or ehealth* or electronic health* or e-technolog* or etechnolog* or digital health* or digital technolog* or mobile health* or mhealth* or m-health* or e-learning* or elearning* or e-medicine* or emedicine* or e-therap* or etherap* or health information technolog* or e-support*).tw,kf. | 28023 |
| 4 | (*Telecommunications/ or *Computers/ or *computers, handheld/ or *microcomputers/ or "User-computer interface"/ or *Internet/) and (Learning/ or Health education/ or Self care/ or Self help groups/ or *Teaching materials/ or *Health knowledge, attitudes, practice/ or *communication/ or *counseling/ or *consumer health information/ or *Therapy, computer assisted/ or *health services accessibility/ or *delivery of health care/) | 8619 |
| 5 | ((online or on-line or digital* or electronic or computer* or software or internet* or web or website* or technology-based or interactiv* or telecommunicat* or "information and communication technolog*" or ICT) adj3 (support or self help or chat or communicat* or self care or self manag* or self efficac* or intervention* or education* or training or learning or teaching or health information* or information service* or lifestyle* or life style* or motivat* or healthcare or health care or complian* or adheren* or monitor* or delivery or behaviour* or behavior* or health promotion* or home health* or home care or counsel* or decision or tool*)).tw,kf. | 73683 |
| 6 | or/1-5 | 150914 |
| 7 | healthy lifestyle/ or healthy diet/ or sedentary lifestyle/ | 9541 |
| 8 | Snacks/ or Health Promotion/ or Feeding Behavior/ or Food Preferences/ or Diet/ | 279139 |
| 9 | Fruit/ or Vegetables/ | 50687 |
| 10 | *"diet, food, and nutrition"/ or meals/ or breakfast/ or lunch/ or snacks/ | 4278 |
| 11 | Exercise/ | 94461 |
| 12 | body weight/ or body weight changes/ or overweight/ or obesity/ or obesity, morbid/ or pediatric obesity/ | 353078 |
| 13 | (healthy eating or heathy life style or food behaviour or diet* or nutrition or health promotion or food preferenc* or lunch* or snack* or meal* or sedentary life style or sedentary lifestyle or fruit* or vegetabl* or feeding behavio?r or physical activity or exercise or sport* or walking* or runnig* or jogging or obesity or overweight or body mass index or BMI or weight*).tw,kf. | 2107101 |
| 14 | or/7-13 | 2317809 |
| 15 | exp Australia/ or (Australia* or Queensland* or New south wales or victoria* or tasmania* or northern territory*).tw,kf. | 187413 |
| 16 | New Zealand/ or New Zealand*.tw,kf. | 65799 |
| 17 | exp Canada/ or (canad* or alberta* or british columbia* or manitoba* or new brunswick* or newfoundland* or labrador* or northwest territories or nova scotia* or nunavut* or ontario* or prince edward island* or quebec* or saskatchewan* or yukon territory*).tw,kf. | 214862 |
| 18 | exp United States/ or (united states or North america* or USA).tw,kf. | 1479738 |
| 19 | (Alabama* or Alaska* or Arizona* or Arkansas* or California* or Colorado* or Connecticut* or Delaware* or Florida* or Georgia* or Hawaii* or Idaho* or Illinois* or Indiana* or Iowa* or Kansas* or Kentucky* or Louisiana* or Maine* or Maryland* or Massachusetts* or Michigan* or Minnesota* or Mississippi* or Missouri* or Montana* or Nebraska* or Nevada* or New Hampshire* or New Jersey* or New Mexic* or New York* or North Carolina* or North Dakota* or Ohio* or Oklahoma* or Oregon* or Pennsylvania* or Rhode Island* or South Carolina* or South Dakota* or Tennessee* or Texas or Texan* or Utah* or Vermont* or Virginia* or Washington* or West Virginia* or Wisconsin* or Wyoming*).tw,kf. | 392119 |
| 20 | exp Great Britain/ or (Britain* or British or United Kingdom* or England* or English or Scotland* or Scottish* or Wales or Welsh or Channel Islands or Northern Ireland* or Irish).tw,kf. | 575806 |
| 21 | "scandinavian and nordic countries"/ or denmark/ or greenland/ or finland/ or iceland/ or norway/ or svalbard/ or sweden/ or (Scandinavia* or nordic or denmark* or danish or greenland* or finland* or finnish or iceland* or norway* or norwegian* or sweden* or swedish).tw,kf. | 267110 |
| 22 | austria/ or belgium/ or france/ or germany/ or monaco/ or netherlands/ or Switzerland/ or Greece/ or Italy/ | 411795 |
| 23 | (Belgium or France or Monaco or Netherlands or Austria or Germany or Switzerland or Greece or Italy).tw,kf. | 261938 |
| 24 | or/15-21 | 2781349 |
| 25 | (randomized controlled trial or controlled clinical trial or clinical study).pt. or random*.tw. or clinical trials as topic/ or trial.ti. | 1392701 |
| 26 | Review.pt. or (systematic review* or meta-analysis or metaanalysis).ti. | 2497050 |
| 27 | Epidemiologic studies/ or cohort studies/ or case control studies/ or follow-up studies/ or longitudinal studies/ or prospective studies/ or retrospective studies/ or Cross-Sectional Studies/ | 2204103 |
| 28 | (observational study or Evaluation studies).pt. or observational studies as topic/ or evaluation studies as topic/ | 415619 |
| 29 | (epidemiologic* or ecological* or experimental or cohort or evaluation or pre-post or quasi* or nested or follow-up or longitudinal* or prospective* or controlled or retrospective* or nonrandomi?ed or case-control or cross-sectional or population).tw,kf. | 5833223 |
| 30 | or/25-29 | 9117810 |
| 31 | 6 and 14 and 24 and 30 | 2920 |
| 32 | limit 31 to (english language and yr="2013 -Current") | 1949 |

Objective two: Database(s): Ovid MEDLINE(R) and Epub Ahead of Print, In-Process & Other Non-Indexed Citations, Daily and Versions(R) 1946 to October 19, 2018
Search Strategy:

| **#** | **Searches** | **Results** |
| --- | --- | --- |
| 1 | Mobile applications/ or *Webcasts as topic/ or *social networking/ or *smartphone/ or *text messaging/ or video games/ | 11809 |
| 2 | (webcast* or podcast* or mobile application* or smartphone* or iphone or android or text messag* or app or apps or virtual reality or ipad* or tablet comput* or tablet device* or instant messag* or SMS* or whatsapp or Youtube* or video gam*).tw,kf. | 51751 |
| 3 | *Telemedicine/ or *remote consultation/ or *remote sensing technology/ or *videoconferencing/ | 20015 |
| 4 | (e-health* or ehealth* or electronic health* or e-technolog* or etechnolog* or digital health* or digital technolog* or mobile health* or mhealth* or m-health* or e-learning* or elearning* or e-medicine* or emedicine* or e-therap* or etherap* or health information technolog* or e-support*).tw,kf. | 27983 |
| 5 | (Telemedicine or telehealth* or telecare* or telemonitor* or remote consult* or remote monitor* or televideo* or teleconsult* or e-consult* or econsult* or telemedical* or remote sensing technolog* or teleconferenc* or video conferen* or videoconferenc*).tw,kf. | 18764 |
| 6 | (*Telecommunications/ or *Computers/ or *computers, handheld/ or *microcomputers/ or "User-computer interface"/ or *Internet/) and (Learning/ or Health education/ or Self care/ or Self help groups/ or *Teaching materials/ or *Health knowledge, attitudes, practice/ or *communication/ or *counseling/ or *consumer health information/ or *Therapy, computer assisted/ or *health services accessibility/ or *delivery of health care/) | 8618 |
| 7 | ((online or on-line or digital* or electronic or computer* or software or internet* or web or website* or technology-based or interactiv* or telecommunicat* or "information and communication technolog*" or ICT) adj3 (support or self help or chat or communicat* or self care or self manag* or self efficac* or intervention* or education* or training or learning or teaching or health information* or information service* or lifestyle* or life style* or motivat* or healthcare or health care or complian* or adheren* or monitor* or delivery or behaviour* or behavior* or health promotion* or home health* or home care or counsel* or decision or tool*)).tw,kf. | 73611 |
| 8 | or/1-7 | 171727 |
| 9 | healthy lifestyle/ or healthy diet/ or sedentary lifestyle/ | 9537 |
| 10 | Snacks/ or Health Promotion/ or Feeding Behavior/ or Food Preferences/ or Diet/ | 279093 |
| 11 | Fruit/ or Vegetables/ | 50677 |
| 12 | *"diet, food, and nutrition"/ or meals/ or breakfast/ or lunch/ or snacks/ | 4276 |
| 13 | Exercise/ | 94446 |
| 14 | body weight/ or body weight changes/ or overweight/ or obesity/ or obesity, morbid/ or pediatric obesity/ | 353039 |
| 15 | (healthy eating or heathy life style or food behaviour or diet* or nutrition or health promotion or food preferenc* or lunch* or snack* or meal* or sedentary life style or sedentary lifestyle or fruit* or vegetabl* or feeding behavio?r or physical activity or exercise or sport* or walking* or runnig* or jogging or obesity or overweight or body mass index or BMI or weight*).tw,kf. | 2105905 |
| 16 | or/9-15 | 2316601 |
| 17 | family/ or parents/ or fathers/ or mothers/ or single parent/ or siblings/ or single-parent family/ or parenting/ or grandparents/ | 182076 |
| 18 | (family* or families or parent* or father* or mother* or paternal* or maternal* or grandparent* or sibling* or caregivers or "care givers" or teacher*).tw,kf. | 1559352 |
| 19 | adults/ or Child/ or Adolescent/ or (Child* or Adolescen* or Teenag* or adult*).tw,kf. | 6885256 |
| 20 | or/17-19 | 7797236 |
| 21 | FEASIBILITY STUDIES/ | 59819 |
| 22 | (functionalit* or feasibilit* or accept* or marketing or promoting or access* or appeal* or evaluat* or validity or validat* or reliability*).tw,kf. | 4482368 |
| 23 | (user* adj2 (test* or engag* or satisf* or opinion* or feedback or review* or explor* or navigat* or thought* or integrat*)).tw,kf. | 6256 |
| 24 | or/21-23 | 4495969 |
| 25 | exp Australia/ or (Australia* or Queensland* or New south wales or victoria* or tasmania* or northern territory*).tw,kf. | 187329 |
| 26 | New Zealand/ or New Zealand*.tw,kf. | 65778 |
| 27 | exp Canada/ or (canad* or alberta* or british columbia* or manitoba* or new brunswick* or newfoundland* or labrador* or northwest territories or nova scotia* or nunavut* or ontario* or prince edward island* or quebec* or saskatchewan* or yukon territory*).tw,kf. | 214772 |
| 28 | exp United States/ or (united states or North america* or USA).tw,kf. | 1479476 |
| 29 | (Alabama* or Alaska* or Arizona* or Arkansas* or California* or Colorado* or Connecticut* or Delaware* or Florida* or Georgia* or Hawaii* or Idaho* or Illinois* or Indiana* or Iowa* or Kansas* or Kentucky* or Louisiana* or Maine* or Maryland* or Massachusetts* or Michigan* or Minnesota* or Mississippi* or Missouri* or Montana* or Nebraska* or Nevada* or New Hampshire* or New Jersey* or New Mexic* or New York* or North Carolina* or North Dakota* or Ohio* or Oklahoma* or Oregon* or Pennsylvania* or Rhode Island* or South Carolina* or South Dakota* or Tennessee* or Texas or Texan* or Utah* or Vermont* or Virginia* or Washington* or West Virginia* or Wisconsin* or Wyoming*).tw,kf. | 391979 |
| 30 | exp Great Britain/ or (Britain* or British or United Kingdom* or England* or English or Scotland* or Scottish* or Wales or Welsh or Channel Islands or Northern Ireland* or Irish).tw,kf. | 575683 |
| 31 | "scandinavian and nordic countries"/ or denmark/ or greenland/ or finland/ or iceland/ or norway/ or svalbard/ or sweden/ or (Scandinavia* or nordic or denmark* or danish or greenland* or finland* or finnish or iceland* or norway* or norwegian* or sweden* or swedish).tw,kf. | 266999 |
| 32 | austria/ or belgium/ or france/ or germany/ or monaco/ or netherlands/ or Switzerland/ | 317044 |
| 33 | (Belgium or France or Monaco or Netherlands or Austria or Germany or Switzerland).tw,kf. | 201038 |
| 34 | or/25-33 | 3147737 |
| 35 | 8 and 16 and 20 and 24 and 34 | 1857 |
| 36 | (letter or note or comment or editorial or legal cases or legislation or news or newspaper article or patient education handout).pt. | 1874736 |
| 37 | 35 not 36 | 1852 |
| 38 | limit 37 to (english language and yr="2008 -Current") | 1567 |

Objective three: Searches executed in Google Advance Search (search terms)

|  | ‘All of these words’ | ‘Any of these words’ |
| --- | --- | --- |
| Search 1 | lunch box | ideas, tips, health, school |
| Search 2 | school lunch box | tips, ideas, healthy |
| Search 3 | lunch | health, ideas, tips, school |
| Search 4 | lunch box | planner, planning tool |

*Note*: Each search was limited to English and repeated in Australia, NZ, UK, Canada, USA (Total 20 searches) and the first 10 pages (100 results) of each search were screened

Searches executed within Apple Store (search terms and number of results)

| Search term | n | Search term | n | Search term | n | Search term | n | Search term | n |
| --- | --- | --- | --- | --- | --- | --- | --- | --- | --- |
| Lunch box | 50 | Packed lunch | 50 | Nutrition | 50 | Family meals | 50 | Lunch ordering | 50 |
| Lunchbox | 50 | School lunch | 50 | Nutrition education | 50 | School meals | 38 | canteen | 50 |
| Lunch box ideas | 50 | Lunch | 50 | Nutrition information | 50 | Children lunch | 12 | Tuck shop | 9 |
| Healthy lunch box | 10 |  |  | Healthy eating | 50 |  |  | Snacks | 50 |
