# Supplementary material for: A systematic evaluation of digital nutrition promotion websites and apps for supporting parents to influence children’s nutrition
Source: Int J Behav Nutr Phys Act. 2020 Feb 10;17:17. doi: 10.1186/s12966-020-0915-1 (PMC7011240; doi:10.1186/s12966-020-0915-1)
Supplement: Supplementary file 2 — Additional file 2. Data extraction table for studies evaluating effectiveness of digital health platforms for improving nutrition outcomes. Table of data extracted from included studies regarding the effectiveness of the digital health platforms [file 12966_2020_915_MOESM2_ESM.docx]

Additional File 2 - Data extraction table for studies evaluating effectiveness of digital health platforms for improving nutrition outcomes

| **STUDY** | **INTERVENTION** | **STUDY DESIGN** | **OUTCOMES** | | |
| --- | --- | --- | --- | --- | --- |
|  |  |  | **KNOWLEDGE / ATTITUDES** | **SELF-EFFICACY / OTHER** | INTAKE & ANTHROPOMETRIC |
| **Study 1**  **Delisle Nystrom et al 2017 (38)**  **Study 2**  **Delisle Nystrom et al 2018 (39)**  **Sweden**  **Quality rating:** Strong | **Platform:** App  **Description:** 6mth MINISTOP app - information to support healthy eating/PA in preschool-aged children, to prevent obesity, content delivered bi-weekly, contact time not reported  **Components:** Information, push notifications, parent tracking of child behaviours, feedback on behaviours, access to dietitian and psychologist | **Participants:** Parents of 4yo children (S1 n=315, S2 n=263)  **Study design:** RCT  **I:** MINISTOP app  **C** Pamphlet on healthy eating/ PA  **OUTCOMES (child):**   - Fat mass index (FMI) - PA and sedentary time (accelerometer) - FV, lollies, SSB intake (questionnaire) - Composite score: Total FMI, PA, sedentary time, FV, lollies, SSB | --- | --- | **Overall effectiveness:** Mixed results**;** Not maintained at 12-months  **S1: 6 months**   - I vs C improved 7-item composite score (*p=*0.021) [I: +0.36units, C: -0.07 units; greater ↑ if higher FMI] - I vs C ↑ odds for improving 6 behaviours (excl. FMI) [OR: 1.99; 95%CI: 1.20-3.30; *p=0.008*] - I vs C sig. ↓ mean intake of SSB (*p*=0.049) [I: -12ml/d, C: +8ml/d] - I vs C no difference change in FMI, PA, sedentary time, FV, lollies   **S2: 12 months**   - No maintained effect for difference in composite score I vs C - I vs C no difference change in FMI, PA, sedentary time, FV, lollies, SSB |
| **Grimes et al 2018 (32)**  **Australia**  **Quality rating:** Moderate | **Platform:** Website  **Description:**  DELISH (Digital Education to Limit Salt Intake in the Home) – Web-based Nut Ed to ↓ salt intake in school children, content delivered weekly, contact time ~23 mins per session  **Components:**  Web-based interactive sessions: games, videos, goal setting, access to website, newsletters, text messages | **Participants:** Child & parent (n=83), Child mean age 9.2y  **Study design:** Pre/Post  **I:** 5-week DELISH Nut Ed  **C:** Nil  **OUTCOMES (child):**  Salt knowledge, attitudes, self-efficacy (questionnaire)  Salt intake (Questionnaire, urinary excretion) | **Overall effectiveness:** Improved knowledge; no effect on attitudes  **Knowledge:**   - ↑ Overall knowledge (↑3.6 units, *p<0.001*; Cohen’s δ=1.16) - ↑ Awareness of daily salt intake (pre 44%, post 72%, *p=0.001)* - ↑ Correct identification of sodium-content on food label (pre 52% post 91%, *p<0.001*).   **Attitudes:**   - No change in attitudes to salt   ( *p>0.05*) | **Overall effectiveness:** Improved self-efficacy   - ↑ Overall self-efficacy (↑0.9 units, *p<0.001*; Cohen’s δ=0.64*)*   . | **Overall effectiveness:** Mixed results   - No sig change in salt intake from pre (5.4 g/day) to post (5.3 g/day) (*p*>0.05) or % exceeding UL (*p*>0.05) - Improvement in salt behaviour (use) score (↑1.3 units, *p*<0.001, Cohens δ 1.08). - 19%↓ placing salt shaker on table during meals (*p*<0.002). - 8% ↓ use of salt at the table (p>0.05) |
| **Study 1**  **Knowlden et al 2015 (35)**  **Study 2 Knowlden et al 2016 (33)**  **Study 3 Knowlden et al 2018 (34)**  **USA**  **Quality rating:** Moderate | **Platform:** Website  **Description:** EMPOWER - Maternal childhood obesity prevention program, 4 weeks, content delivered weekly, contact time ~30 mins per session  **Components:**  5 modules (one per each behaviour outcome): audiovisual presentation, interactive worksheets, discussion board posts. | **Participants:** Mothers of 4-6 yo child (S1, S2 n=57, S3 n=37)  **Study design:** RCT  **I:** EMPOWER website (n=29)  **C:** Healthy lifestyle information delivered online (n=28)  **OUTCOMES:**  Online questionnaire:   - Mother’s self-efficacy - Child intake of FV & Sugar-free beverages (SFB) | **--** | **Overall effectiveness: N**o effect on self-efficacy  **Study 1 (8weeks):**   - No effect self-efficacy for FV, SFB in I or C - I vs C sig ↑ FV outcome expectations; home availability; No sig. effect SFB   **Study 2 (1 year):**  - No effect self-efficacy for FV, SFB in I or C  **-** I vs C sig ↑ FV home availability; No sig. effect SFB  **Study 3 (2 year):**  - No effect self-efficacy for FV, SFB in I or C  - I vs C sig ↑ FV home availability; No sig. effect SFB | **Overall effectiveness:** Sig. intervention effect on FV, increase in SFB in both groups; All maintained at year 2.  **Study 1 (8 weeks):** -FV intake: I vs C sig ↑ of 1.61cups FV (95% CI: 0.698- 2.529; *p*<0.001); Group x time effect (*p=0.036*; Cohen’s *f* = 0.160)   - SFB intake: Sig. ↑ both groups of 1.070 cups (95% CI: 0.324- 1.816; *p=*0.003)   **Study 2 (1 year):**   - FV intake: I vs C sig ↑ of 1.847cups (95% CI: 1.20- 2.49; *p*<0.001); Small group x time effect (*p=0.012*; Cohen’s *f* = 0.210) - SFB intake: Sig. ↑ in both groups of effect size (*p*=0.002; Cohen’s *f* =.275)   **Study 3 (2 years):**  - FV intake: I vs C sig ↑ 1.690 cups FV (95% CI: 1.11-2.25, p<0.01). Group x time effect (*p=0.033*; Cohen’s *f* = 0.139)  - SFB intake: Sig. ↑ in both groups of effect size (*p<0.001*; Cohen’s *f* =0.321) |
| **Rangelov et al 2018 (36)**  **Switzerland**  **Quality rating:** Weak | **Platform:** Website  **Description:**  ‘Famiglia, Attività fisica, Nutrizone’ (FAN) Social Marketing program to promote healthy food consumption & regular physical activity, 8 week program, content delivered weekly, contact time not reported  **Components:**  Parents receive via website, children receive letter via post. Website: advice, recipes, videos, forum with dietitian, email/SMS weekly reminders. | **Participants:** Children in grades 1-2 & parents (n=608)  **Study design:** RCT  **I1:** Web + SMS (G3)  **I2:** Web + email (G2)  **C:** Web only (G1)  **OUTCOMES:**  Child intake of FV, sweets (7-day food diary) | --- | --- | **Overall effectiveness:** Mixed/positive effect on fruit, vegetable, sweets in all groups  **Fruit intake**   - ↑F from BL to 8wks in all groups, but sig. only for G1 (*r*=0.17; SE=0.09; *p<0.05)*   **Sweets intake**   - ↓ sweets from BL to 8wks in all groups, but sig. only for G1 (*r*=-0.11, SE=0.05) *p<0.05)*   **Vegetable intake**   - ↑V from BL to 8wks in G3 (*r*=0.12; SE=0.06; *p<0.05)* - No change G1, G2 |
| **De Lepeleere et al 2017 (30)**  **Belgium**  **Quality rating:** Weak | **Platform:** Website  **Description:**  Movie Models – health promoting online video intervention, 4 weeks, content delivered weekly, contact time ~2 mins per video  **Components:**  22 videos on difficult parenting situations (PA, ST, healthy diet -water, BF). | **Participants:** Parents of primary school children **(**n=238)  **Study design:** Quasi-experimental  **I:** Movie Models  **C:** Waitlist control  **OUTCOMES:**  Parent questionnaires at BL, 1-mth (T1), 3mth (T2):  Parent self-efficacy;  Parenting practices & feeding strategies; Child FV, water, SSB, snack intake (FFQ) | **---** | **Overall effectiveness:** I vs C positive effect on self-efficacy   - I vs C: ↑ self-efficacy motivating child to eat V (F=4.95, *p=0.03*), giving child freedom to drink water (F=6.07, *p=0.003*), ↓ for having V available (F=4.79, *p=0.03*).   Only sig. effects shown among parents of younger children (6-9yo):   - T1: I vs C: ↑ self-efficacy for letting child choose different F (F=8.38; p=0.005), and V availability (F=5.80, *p*=*0.02*) - T2: I vs C: ↑ self-efficacy for involving children in buying V (F=5.56; *p=0.02*). | **Overall effectiveness:** No effect on behaviours, some +ve effects on parenting practices at T2  **Child behaviours:**   - I vs C no sig. effect on child intake   **Parenting practices:**   - I vs C no sig. effect on parenting practices concerning food intake at T1 or T2, except: small effect I vs C on ‘motivating child to eat fruit’ at T2 (F=8.00; *p=0.006*) - I vs C sig. effect in parents of older children (10-12yo): ↑ permissiveness of how much V child allowed to eat b/w meals at T2 (*F = 11.70; p < 0.001*) |
| **Au et al 2016 (29)**  **USA**  **Quality rating:** Moderate | **Platform:** Website  **Description:** Online Nut Ed for Special Supplemental Nutrition Program for Women, Infants, and Children (WIC) participants, once off session, contact time ~15-20 mins  **Components**: Online BF lessons: importance of daily BF, health outcomes of skipping BF, BF preparation, goal-setting | **Participants:** WIC participants - mothers of 1-5yo children (n=590)  **Study design:** RCT  **I:** Online Nut Ed (n=231)  **C:** In-person group Nut Ed (n=359)  **OUTCOMES:**  Parent questionnaire at 2-4mth:BF Knowledge; Attitudes (skipping BF); Self-efficacy (BF intake & preparation); BF intake (child & parent) | **Overall effectiveness:** Intervention effect on improved attitudes (barriers), improved knowledge both groups  **Knowledge:**   - I & C both sig. ↑ knowledge (*p<0.05*); Greater magnitude change in C vs I for sugar on cereal knowledge)   **Attitudes:**   - I vs C ↓ barriers for skipping BF (*p*≤0.02): lack of time, not enough food at home, hunger, difficulty with preparation | **Overall effectiveness:** Improved self-efficacy both groups, somewhat greater effect in I vs C   - I & C ↑ self-efficacy for F at BF (*p*<0.05) - I sig ↑ self-efficacy (*p*<0.05) for child BF each day, offering WIC foods at BF, but not sig. greater ↑ compared with C | **Overall effectiveness:** Significant Intervention effect on BF intake  **Parent BF intake:**   - I vs C sig. greater ↑ freq BF *(p=0.0007)*; no effect freq. intake BF with child   **Child BF intake**   - I vs C sig. greater ↑ freq eat something at BF *(p=0.01)*; no effect freq. intake BF with child - I & C sig. ↑ eat breakfast of WIC foods, eat breakfast at childcare *(p<0.05);* no sig. diff. b/w groups |
| **Wilson et al 2014 (37)**  **USA**  **Quality rating:** Weak | **Platform:** Website  **Description:** Web-based tailored parenting tailored intervention to increase FV intake, once-off module, contact time predicted ~45-60 minutes  **Components:** Tailored online feedback & messages based on survey data | **Participants:** Parents & adolescents (n=41)  **Study design: P**re/Post  **I:** Website/ tailored feedback  **C:** Nil  **OUTCOMES (at 1wk):**  FV intake screening tool | --- | --- | **Overall effectiveness:** Significant positive effect on FV intake  **Fruit intake (serves per day)**   - Parents ↑ F (1.76±1.02 vs 2.32±1.23 serves; *p*<*0.01)* - Adolescents ↑ F (1.71±0.93 vs 2.27±0.92 serves; *p<0.05*)   **Vegetable intake (serves per day)**   - Parents ↑ V (3.68±1.47 vs 4.39±2.07 serves; *p<0.05*) - Adolescents ↑ V (3.34±1.46 vs 4.07±1.47; *p*<0.05) |
| **Delamater et al 2013 (31)**  **USA**  **Quality rating:** Weak | **Platform:** Website  **Description:**  FIT-4-Health (Families Interacting Together for Health) – family program for overweight children, website as information source, contact time not reported  **Intervention:**  Obesity/healthy lifestyle information, Ax of diet/PA, interactive game, goal-setting | **Participants:** Families with overweight children aged 8-12y (n=24)  **Study design:** Pre/Post  **I:** FIT-4-Health for 4wks  **C:** n/a  **OUTCOMES (child):**  Self-efficacy: Intrinsic Motivation Inventory (IMI); BMI z-score (measured), Food intake, PA & sedentary behaviour (Ques); Healthy lifestyle score (based on food intake, PA, sedentary) | --- | **Overall effectiveness:** Positive effect on self-efficacy   - Sig. ↑ in self-efficacy at F/UP *(p*=0.025). - High users ↑ more over time vs low users (F = 2.86, *p*=0.06). | **Overall effectiveness:** Positive effect on healthy lifestyle score. Positive effect on food intake & BMI for high users only  **Intake**   - No sig. ↑ food intake at F/UP - High users sig. ↑ over time relative to low users (F= 3.37, *p=*0.04)   **Healthy lifestyle score**   - Sig ↑ in score at F/UP (*p=0.001)* - High users sig. ↑ over time relative to low users (F =16.61, *p*=0.001)   **BMI**   - No sig. change in z-BMI at F/UP - High users ↓ *z*-BMI, low users ↑*z-*BMI over time (F=6.36, *p*=0.02) |

**Abbreviations**: App = mobile application; Avg. = Average; Ax = assessment; b/w = Between; B = Baseline; BF = breakfast; BL = baseline C = Control; CI = Confidence Interval; F/UP = Follow up; FFQ = Food frequency questionnaire; Freq = frequency; F = Fruit; FMI = fat mass index; HI = High-income; I = Intervention; LI = Low-income; M = Mean; No. = number; Nut Ed = nutrition education; PA = Physical Activity; RCT = Randomised Control Trial; SB = sweetened beverage; SD = Standard Deviation; SE = standard error; SES = Socio-Economic Status; SFB = sugar-free beverage; Sig. = Significantly/Significant; SSB = Sugar-sweetened beverage; ST = screen time; T = time; UL = Upper Limit; USA = United States of America; V = Vegetables; WIC = Women Infants and Children; y = years old; ↑ = Higher/increase; ↓ = Lower/decrease; -ve = Negative; +ve = Positive; /d = days; /wk = week; /mth = month
