# Supplementary material for: A systematic evaluation of digital nutrition promotion websites and apps for supporting parents to influence children’s nutrition
Source: Int J Behav Nutr Phys Act. 2020 Feb 10;17:17. doi: 10.1186/s12966-020-0915-1 (PMC7011240; doi:10.1186/s12966-020-0915-1)
Supplement: Supplementary file 3 — Additional file 3. Characteristics and outcomes of user-testing studies (objective two). Table of study characteristics and outcomes of user-testing from included studies [file 12966_2020_915_MOESM3_ESM.docx]

Additional File 3: Characteristics and outcomes of user-testing studies (Objective two)

| **STUDY** | **INTERVENTION** | **STUDY CHARACTERISTICS** | **RESULTS** |
| --- | --- | --- | --- |
| **Biediger-Friedman et al 2018 (42)**  **United States of America** | **Platform**: App ‘WIC’  **Purpose:** Collect preferences from WIC participants regarding nutrition education app.  **Components:** Tracking/locating, accessing information, chatting/texting, gaming, planning/scheduling, sharing  **Target audience^a^:** Mothers involved in WIC program | **Study design:** Focus groups, survey  **Participants:** n=48 mothers at WIC clinics in South-Central Texas  **OUTCOMES:** Preferred mode of delivery, preferred features, content & technology | **Preferred mode of delivery:**   - All had smartphone and predicted app usage would be frequent (few times/week or /day)   **Preferred features:**   - Self-explanatory and easy to use - Targeting multiple behaviours - Receiving nutrition education through videos - Logging and tracking features - Live interface (staff chat options for support) - Sharing information through social media - Sharing milestones, ideas, achievements, challenges - Games to engage children - Features that engaged whole family   **Content**:   - Trusted, beneficial, accurate and relevant   **Technology:**   - Motivational prompts (challenges, pings, reminders, celebratory signals) |
| **Luesse et al 2018 (45)**  **United States of America** | **Platform**: Text messaging linked to website  **Purpose:** Determine if text messaging is a suitable way to reach parents with food & dietary information  **Components:** N/A  **Target audience^a^:** Low income urban minority families | **Study design:** Focus groups  **Participants:** n=16 low income parents of elementary school children  **OUTCOMES:** Preferred mode of delivery, content & technology | **Preferred mode of delivery:**   - E-mails, website, text-messages, social media - Provided across platforms (link to website in message) - Infrequent (monthly) written information as adjunct to website - Provided from a figure outside of the family   **Content:**   - Tailored and personalised messages - Preferred themed over general advice - Pictures for low-literacy parents - Visual and written content preferred   **Technology**:   - Some didn’t like idea of receiving text messages, others thought would be helpful - Evening most preferred time to receive texts |
| **Reynolds et al 2018 (20)**  **Australia** | **Platform**: App  **Purpose:** Evaluation of school-based mobile app to deliver messages to parents on how to pack a healthy lunchbox  **Components:** N/A  **Target audience^a^:** Principals Parents of primary-school aged children | **Study design:** Survey  **Participants:** n=196 primary school principals  **OUTCOMES:** Preferred mode of delivery, preferred features | **Preferred mode of delivery:**   - 60% currently used app - >80% agree appropriate for schools to provide information though app - Most considered at least 1/month as acceptable frequency for messages to be sent - 73% agreed it would be acceptable for these messages to be provided by a third party - 3 ceased use of apps as they didn’t enhance communication with parents   **Preferred features:**   - Of those who had not previously used a school-based app, 33% were likely to use in future - Cost & communication most influential features |
| **Hull et al 2017 (44)**  **United States of America** | **Platform**: App ‘Children Eating Well’ (CHEW)  **Purpose:** Home-based nutrition education intervention to reinforce Special Supplemental Nutrition Program for WIC  **Components:**  Shopping tools (barcode scanner, calculator), nutrition education (yummy snack gallery, recipes, snacking tips)  **Target audience^a^:** Low income mothers of children <5 years | **Study design:** Questionnaire  **Participants:** n=63 mothers of African-American & Hispanic children 2-4 years  **OUTCOMES:** Preferred features, technology & useability/appeal | **Preferred features:**   - Snack gallery was helpful, loved by children, easy, affordable - Shopping tools - Tips to help buy/eat more fruit & vegetables   **Disliked features:**   - Not enough recipes   **Technology:**   - Some disliked notification delivery schedule or information provided through notifications   **Useability/appeal:**   - Snack gallery/healthy snack tips rated high for ease of use, helpfulness, usefulness, satisfaction - Barcode scanner & produce calculators rated high for ease of use and helpfulness - Shopping tools rated high for satisfaction |
| **Wyse et al 2017 (21)**  **Australia** | **Platform**: Canteen website  **Purpose:**  Evaluate online canteen ordering systems  **Components:** N/A  **Target audience^a^:** Parents and students | **Study design:** Survey  **Quality rating^b^:** N/A  **Participants:** n=123 primary school principals  **OUTCOMES:** Perceived barriers to implementation | **Perceived barriers to implementation:**   - Parent internet access (63%) - Set up time (52%) - Canteen internet access (41%) - Difficulty of use for parents (39%) - Difficulty of use for canteen manager (35%) |
| **Avis et al 2016 (41)**  **Canada** | **Platform**: App (iPad) ‘Resource Information Program for Parents on Lifestyle and Education’ (RIPPLE)  **Purpose:**  e-Health program for parents to prevent childhood obesity in primary care  **Components:**  Screening (weight status), brief intervention (tailored feedback), referral to treatment (resources, community services)  **Target audience^a^:** Parents, health care professionals, researchers | **Study design:** Focus groups  **Participants:** n=20 paediatric healthcare professionals, n=10 parents, n=8 researchers and graduate trainees  **OUTCOMES:** Preferred mode of delivery, preferred features, content & useability/appeal | **Preferred mode of delivery:**   - Usability of iPad was straightforward   **Preferred features:**   - Quick, informative, tailored feedback - Alleviates barriers & motivates to make changes - Relevant resources - Awareness of child weight status, dietary, physical activity & sedentary behaviours - Initiates conversation with paediatrician   **Disliked features:**   - Eliciting negative reactions (fear, guilt, shame) - Content in e-mail report was vague   **Content:**   - Want additional resources - Improve weight related terminology - Appropriate language, suitable in length   **Useability/appeal:**   - More clarity re. instructions, descriptions, terms - Images should reflect cultural diversity |
| **Biediger-Friedman et al 2016 (43)**  **United States of America** | **Platform**: App  **Purpose:**  User-centred design of app to improve health behaviours among participants in the Special Supplemental Nutrition Program for WIC  **Components:** N/A  **Target audience^a^:** WIC participants | **Study design:** Focus groups  **Participants:** n=64 WIC/WIC-eligible women  **OUTCOMES:** Preferred features & technology | **Preferred features:**   - Connecting with other mothers for support - Sharing health-related data (exercise logs) - Access to professionals for assistance - online support with live chat option - Games for child engagement - Trackers for diet, exercise & breastfeeding - Shopping lists - Customisable home page - All features in one place   **Technology:**   - Library feature with search function - Locators for farmers markets |
| **Burrows et al 2015 (46)**  **Australia** | **Platform**: Website & app  **Purpose:**  Development of an eHealth family healthy lifestyle program  **Components:** N/A  **Target audience^a^:** Parents of children 4-18 years | **Study design:** Survey  **Participants:** n=75 parents of children 4-18 years  **OUTCOMES:** Preferred mode of delivery, preferred features, content, technology & useability/appeal | **Preferred mode of delivery:**   - 90% interested in online lifestyle program - Website in addition to app   **Preferred features:**   - Personal user accounts to access information - Informal program with no scheduled sessions - Information from dietitian (face-to-face or online) - Interaction with members & staff (social network or forum) - Inclusive of child with games & activities - Achievable and monitored goals/goal setting - Not solely focussed on weight - Endorsed by university or govt. website   **Content:**   - Specific information & education on portions for different ages, recipes, nutrition - Relevant for all family members - Budget friendly ideas using everyday foods   **Technology:**   - Enter goals via website or smartphone - Reminders via email or SMS   **Useability/appeal:**   - Easy to use, low cost, simple, streamlined |
| **Rangelov et al 2015 (40)**  **Switzerland** | **Platform**: Website ‘Family, Physical Activity, Nutrition’ (FAN)  **Purpose:**  Describe the development process of the 8-week program providing tailored information to parents and children about importance of healthy eating and PA  **Components:** N/A  **Target audience^a^:** Families of elementary and middle school aged children | **Study design:** Focus groups, co-creation activities, post-intervention survey  **Participants:** n=26 parents, n=38 children (focus groups, co-creation activities), n=389 parents, n=370 children (survey)  **OUTCOMES:** Preferred mode of delivery, preferred features | **Preferred mode of delivery:**   - Internet, email, SMS preferred channels - 86.7% interested in health-related programs delivered via website   **Preferred features:**   - Printed materials for children (81% parents and 83.1% children liked the letters) - Info delivered no more than 1/week - Easy access to tailored information - Ways to improve and maintain behaviours - Practical tips (not just theoretical information) - Access to dietary consultant - Quick, cheap, child-friendly, healthy recipes - Videos (94% parents, 64.5% children) - Forum (65.9% parents) - Receiving e-mail and SMS prompts   **Disliked features**:   - Questionnaires, cursive font, heavy theory |

^a^ Target audience of tested platform(s). **Abbreviations:** App = mobile application; WIC = Women Infants and Children
