# Supplementary material for: A systematic evaluation of digital nutrition promotion websites and apps for supporting parents to influence children’s nutrition
Source: Int J Behav Nutr Phys Act. 2020 Feb 10;17:17. doi: 10.1186/s12966-020-0915-1 (PMC7011240; doi:10.1186/s12966-020-0915-1)
Supplement: Supplementary file 4 — Additional file 4. 4.1 - Summary of included websites (objective three); and 4.2 - Summary of included apps (objective three). Tables of characteristics and summary of websites and apps included in review [file 12966_2020_915_MOESM4_ESM.docx]

Additional File 4 - 1: Summary of included websites (Objective three)

| **WEBSITE INFORMATION** | | | **TOP 3 KEY MESSAGES** | | **FUNCTIONALITY** | |
| --- | --- | --- | --- | --- | --- | --- |
| **Healthy Lunch Box CCNSW (47)**  **Weblink:**  [www.healthylunchbox.com.au](http://www.healthylunchbox.com.au)  **Year developed**:  2018  **Last updated**:  2018  **Affiliation:**  Cancer Council New South Wales  **Jurisdiction**:  Australia  **Cost**: Nil | **Purpose:**  Assist parents & children to plan & pack a healthy LB  **Target audience:**  Parents & children  **MARS website quality mean score:** 4.2/5 | 1. Healthy LB contains: B&C, V & salads, meat & alt., dairy, F, water 2. F&V have great nutritional value, help kids learn & play, cancer prevention & cheaper than packaged food 3. Planning & preparing ahead saves time & reduces stress   **Credibility of information**:   - Messages consistent with ADG & explicitly states based on ADG - No explicit mention of engagement with health professionals | | **Key Features**:   - LB builder, 6 categories (B&C, V & salads, meat & alt., dairy, water), to fill 6 sections of LB - Nutrition & serve size information - Recipes/ideas: LB examples, sandwiches, healthy swaps/snacks, salads, sauces & dips - Tips: (1) Adding F & V; (2) Saving Time; (3) Practical packing tips; (4) Starting with small steps (alternatives and swaps); (5) Involving children; (6) Food safety; (7) Recipe & LB videos - Links to further information & videos   **Useability**:   - Search function – limited - Easy to navigate   **Social / Community features:** Share recipes on social media, like, save or share Vimeo videos  **Technical features:** No login required, email/print content  **Limitations:**   - No personalisation in LB builder (e.g. serve size/child age, dietary pref./requirements) - LB serve size is large - Some foods not targeted toward children, not LB friendly - Crossover between food groups | |  |
| **Go For Your Life - Weigh up your lunch (48)**  **Weblink:**  <http://www.goforyourlife.vic.gov.au/hav/articles.nsf/html/index.html>  **Year developed**:  Unknown  **Last updated**:  Unknown  **Affiliation:**  Department of Health, Victoria  **Jurisdiction**:  Australia  **Cost**: Nil | **Purpose:**  Demonstrate to children & adults how different foods add up to make a healthy or not so healthy LB  **Target audience:**  Parents and/or children  **MARS website quality mean score:** 4.0/5 | 1. Balance needed in LB with 3 snacks, a meal & drink 2. Emphasising ‘everyday’ foods & minimising 'sometimes' foods in LB for balance 3. Emphasis on including foods with good nutritional value   **Credibility of information**:   - Messages consistent with ADG - No explicit evidence of engagement with health professionals | | **Key Features**:   - LB builder, 3 categories (mains, snacks, drinks), fill 1 section with a main, 3 sections with snacks, 1 section for a drink - Choices include ‘everyday’ & ‘sometimes’ foods - Feedback provided on choices made - ‘For parents’ & ‘healthy eating’ sections – written information & links   **Useability**:   - Click & drag items into LB builder - Simple, visual & interactive   **Social / Community features:** Send LB via email to multiple addresses  **Technical features:**  No login/account required  **Limitations:**   - Only general advice regarding how to improve lunchbox - Lack of information/recipes - ‘Go for your life! Victoria’ phased out in 2011, no other part of program available on website & links within LB builder broken | |  |
| **Healthier Lunches for Children (49)**  **Weblink:**  <http://www.healthylunch.org.uk/>  **Year developed**:  Unknown  **Last updated**:  Unknown  **Affiliation:**  Developed in partnership with Healthy Schools Programme in the London Borough of Islington  **Jurisdiction**:  United Kingdom  **Cost**: nil | **Purpose:**  Assist schools in improving quality of lunchtime nutrition in their students  **Target audience:**  Children through schools  **MARS website quality mean score:** 3.3/5 | 1. Packed L represent around 1/3 of a child’s daily intake, therefore should provide food in line with Eatwell food profile 2. Healthy L should contain food from each of the 5 food groups 3. Treat foods should be given only occasionally   **Credibility of information**:   - Messages consistent with/based on UK DG (Eatwell referenced) - No explicit evidence of engagement with health professionals | | **Key Features**:   - 3 step programme (L audit, check & revise school policy, take practical measures to raise standards) - Research page with information & links to guidelines - Information regarding how to pack a healthy L - Children’s LB auditing tool (to be completed by child) - Links to resources, policies, L audit tools, quizzes (can personalise)   **Useability**:   - Basic website   **Social / Community features:** Subscribe to email list  **Technical features:** Login required for parts of the website  **Limitations:**   - Children’s auditing tool does not function well | |  |
| **The Zero Waste and Healthy Lunchbox (50)**  **Weblink:**  <http://ecolosante.wixsite.com/ecolosante/home>  **Year developed**:  2016  **Last updated**:  2016  **Affiliation:**  NWRSC Solid Waste Service & various other departments  **Jurisdiction**:  Canada  **Cost**: nil | **Purpose:**  Educate students & parents about how to eat healthy foods, with a focus on environment  **Target audience:**  Children & parents through schools  **MARS website quality mean score:** 3.3/5 | 1. Pack a zero-waste LB to help the environment 2. Pack a healthy LB to help you grow 3. Packing a zero-waste and healthy LB is easy   **Credibility of information**:   - Messages mostly consistent with Canadian DG (no references/links) - Two public health Dietitians involved, contact details provided, credentials unclear | | **Key Features**:   - Video content – zero-waste LB, composting & packing a healthy LB - Audio content – song to sing in class - Powerpoint presentations describing activities for use in class - PDF resources   **Useability**:   - Both English and French content   **Social / Community features:** Nil  **Technical features:** Nil  **Limitations:**   - Lacking specific advice regarding what to pack in a LB - Contains broken links | |  |
| **Healthy Lunch Box (51)**  **Weblink:**  <http://healthylunchbox.com/>  **Year developed**:  2013  **Last updated**:  2017  **Affiliation:**  Individual(s)  **Jurisdiction**:  North America  **Cost**: nil | **Purpose:**  Inform & educate parents & teachers regarding healthy eating in children  **Target audience:**  Parents & teachers  **MARS website quality mean score:** 2.3/5 | 1. Eat foods from the 5 food groups   **Credibility of information**:   - Messages somewhat consistent with North American DG, some incorrect - Dietitians/nutritionists developed content, however unclear of credentials | | **Key Features**:   - 8 children’s games incorporating healthy eating concepts (e.g. drag food items onto the ‘Healthy eating plate’) & food safety - Recipes (N=16) - Blog - Lesson plans for teachers   **Useability**:   - Basic website   **Social / Community features:** Twitter / FB pages  **Technical features:** Nil  **Limitations:**   - Some games do not function - Minimal information (content delivered via games & lesson plans) | |  |
| **Make Healthy Normal - Healthy School Lunch Box (52)**  **Weblink:** <https://www.makehealthynormal.nsw.gov.au/food/healthy-school-lunch-box>  **Year developed**:  Unknown  **Last updated**:  2018  **Affiliation:**  NSW Government Department of Health  **Jurisdiction**:  Australia  **Cost:** nil | **Purpose:**  Encourage lifestyle changes for better health  **Target audience:**  Parents (of school aged children) & children  **MARS website quality mean score:** 3.8/5 | 1. Balanced LB contains a drink & a variety of everyday foods from core food groups for recess & L 2. Go, Grow & Glow - grain foods to GO, P-rich foods to GROW and V to GLOW 3. Pack F for fibre   **Credibility of information**:   - Messages consistent with ADG   Content developed by an Accredited Practising Dietitian (not evident on website but evidence found elsewhere) | | **Key Features**:   - LB builder – 4 categories (GO, GROW, GLOW & FIBRE) to select 5 foods, can personalise or generate random combination - Limit menu items by cuisine / dietary restriction   **Useability**:   - Part of a larger website with other pages, resources, tips, menu plans re healthy eating - Search function   **Social / Community features:** Share content via social media  **Technical features:** Nil  **Limitations:**   - No information/recipes/tips specific to LB provided   Foods in LB builder do not make up a cohesive meal | |  |
| **Healthy Kids Association - Packing a healthy lunchbox (53)**  **Weblink:**  <http://healthy-kids.com.au/parents/packing-a-healthy-lunchbox/0>  **Year developed**:  Unknown  **Last updated**:  Unknown  **Affiliation:**  Non-government organisation  **Jurisdiction**:  Australia  **Cost**: nil | **Purpose:**  Improve nutritional quality of food in schools & at home for children  **Target audience:**  Parents  **MARS website quality mean score:** 3.7/5 | 1. Important to pack balanced L so children are getting the nutrients they need 2. Pack the core 4 + 1 for active kids (a main L, core snack, F, water + additional small reduced fat milk drink/F juice, +1 extra snack for active kids) 3. Keep "occasional" foods out of the LB, keep for special occasions   **Credibility of information**:   - Messages consistent with ADG - No explicit evidence of engagement with health professionals, but partnered with Dietitians Association of Australia | | **Key Features**:   - PDF resource – LB guide based on core 4 + 1 concept - Information re. 5 core food groups & how to choose healthy snacks - 10-day lunchbox menu (pictures only)   **Useability**:   - Part of a larger website with other pages, blogs, resources, recipes - Search function   **Social / Community features:** Subscribe to email list, share 10-day LB menu via social media / email, Twitter, FB, Instagram, Google+, YouTube & Vimeo accounts  **Technical features:** Sign up/create account for access to some content | |  |
| **Nutrition Australia - Healthy lunchbox week (54)**  **Weblink:**  <https://www.healthylunchboxweek.org/>  **Year developed**:  Unknown  **Last updated**:  2018  **Affiliation:**  Nutrition Australia (National initiative)  **Jurisdiction**:  Australia  **Cost:** nil | **Purpose:**  Inspire & empower healthy eating for all Australians  **Target audience:**  Parents of school aged children  **MARS website quality mean score:** 3.6/5 | 1. Make it healthy 2. Keep it convenient 3. Make it enjoyable   **Credibility of information**:   - Messages consistent with ADG - Developed by Nutrition Australia | | **Key Features**:   - Information re. Nutrition Australia Healthy Lunch box Week - Links to resources re. packing a healthy LB, meal ideas, nutrition information, components of a healthy LB   **Useability**:   - Part of a larger website with healthy eating information, recipes - Search function   **Social / Community features:** Share content via social media / email**,** Twitter / FB / Instagram / Linkedin / Youtube pages  **Technical features:** Nil | |  |
| **Healthy Kids NSW - Lunch Box Ideas (55)**  **Weblink:**  <https://www.healthykids.nsw.gov.au/parents-carers/healthy-eating-and-drinking/lunch-box-ideas.aspx>  **Year developed**:  2018  **Last updated**:  2018  **Affiliation:**  Joint initiative NSW Ministry of Health, NSW Department of Education, Office of Sport & NSW Division of the Heart Foundation  **Jurisdiction**:  Australia  **Cost:** nil | **Purpose:**  Support teachers, parents, carers, coaches, health professionals, kids & teens to make healthy choices  **Target audience:**  Parents, children & schools/ teachers  **MARS website quality mean score:** 3.5/5 | 1. Healthy L keeps kids alert & focused, provides them with nutrition they need everyday 2. Pack your child’s L with variety of foods from the five core food groups 3. Don't forget to drink water   **Credibility of information**:   - Messages consistent with ADG - No explicit evidence of engagement with health professionals, but developed in consultation with NSW Ministry of Health and Heart Foundation | | **Key Features**:   - Information regarding LB ideas, how to manage LB refusal - Interactive infographic – 5 ways to a healthy lifestyle - Links to child friendly recipes   **Useability**:   - Part of a larger website with other pages, resources, recipes - Search function   **Social / Community features:** Share content via social media / email  **Technical features:** Nil  **Limitations:**   - Some broken links | |  |
| **Nestle Healthy Active Kids What makes a balanced lunchbox? (56)**  **Weblink:**  <https://www.healthyactivekids.com.au/balanced-lunchbox/>  **Year developed**:  Unknown  **Last updated**:  Unknown  **Affiliation:**  Joint initiative between Australian Institute of Sport and Nestle  **Jurisdiction**:  Australia  **Cost:** nil | **Purpose:**  Encourage Australian primary school students to live a healthy, happy & active lifestyle  **Target audience:**  Children through schools, parents of school aged children  **MARS website quality mean score:** 3.5/5 | 1. Children consume 1/3 of their daily nutrients at school so it's important to pack them a balanced L 2. Balanced L includes variety of foods from the 5 food groups 3. Variety is important for children's growth, development & energy levels   **Credibility of information**:   - Messages consistent with ADG - No explicit evidence of engagement with health professionals | | **Key Features**:   - PDF resource - LB tips & guide - LB lesson plans for schools (by state) - Recipes for LB & snacks - Link to more recipes, videos & other resources   **Useability**:   - Part of a larger website with other pages, resources, recipes, separate sections for parents / teachers / kids - Search function   **Social / Community features:** Nil  **Technical features:** Nil  **Limitations:**   - Some broken links | |  |
| **Healthy Eating Advisory Service - Healthy lunchboxes (57)**  **Weblink:**  <https://heas.health.vic.gov.au/schools/healthy-lunchboxes>  **Year developed**:  Unknown  **Last updated**:  Unknown  **Affiliation:**  Healthy Eating Advisory Service & Nutrition Australia Vic Division  **Jurisdiction**:  Australia  **Cost:** nil | **Purpose:**  Support early childhood services, outside school hours care, schools, workplaces, hospitals, sport & recreation centres, tertiary education & parks to provide healthier foods & drinks  **Target audience:**  Parents of school aged children  **MARS website quality mean score:** 3.4/5 | 1. Pick & mix something from each of the food groups for a healthy LB 2. Include water in LB 3. Sweet & savoury snack foods should be limited in LB   **Credibility of information**:   - Messages consistent with ADG - Developed by Dietitians and Nutritionists via Nutrition Australia | | **Key Features**:   - LB guide - Pick ‘n’ Mix 1-6 poster (1=F, 2=V, 3=Dairy, 4=Meat/alt., 5=B&C, 6=Water), lists examples of what to include - Links to external sites – CCNSW lunch box builder, Nutrition Australia, Better Health Channel - Links to video content from Décor, Nutrition Aus, Dairy Aus, Better Health Channel   **Useability**:   - Part of a larger website containing info for early childhood, schools, workplaces, sport, food outlets, food industry and training - Search function   **Social / Community features:** Share content via social media/email, Twitter / FB / Instagram / Linkedin / Youtube pages  **Technical features:** Increase/decrease font size  **Limitations:**   - Limited information, mostly external links | |  |
| **QLD Education - A healthy start to school tool kit (58)**  **Weblink:**  <https://education.qld.gov.au/initiatives-and-strategies/health-and-wellbeing/student-health-wellbeing/smart-choices/resources/heathly-start-toolkit>  **Year developed**:  Unknown  **Last updated**:  2018  **Affiliation:**  Queensland Government Department of Education  **Jurisdiction**:  Australia  **Cost:** nil | **Purpose:**  Provide information to ensure children starting school are provided with healthy food they need to keep them active & help them concentrate while learning  **Target audience:**  Children via schools, parents of school aged children  **MARS website quality mean score:** 3.3/5 | 1. Smart Choices traffic light system - Green: have plenty (encourage & promote), Amber: select carefully (don’t let dominate the choices, avoid large serves), Red; Occasionally (not to be supplied more than twice per term) 2. L & snacks play big role in providing children daily nutrition 3. Healthy snacks & L give children essential nutrients & energy needed to concentrate   **Credibility of information**:   - Messages consistent with ADG  1. No explicit evidence of engagement with health professionals but likely | | **Key Features**:   - Information re. healthy eating in the school setting, healthy snacks & L, talking to families - Resources re. Smart Choices Healthy Food & Drink Supply Strategy, parent information, toolkits & fact sheets - Links to Department of Education   **Useability**:   - Part of a larger website with an extensive range of pages & links relevant to schools, parents & children - Search function   **Social / Community features:** Twitter / FB / Linkedin / Youtube pages  **Technical features:** Nil | |  |
| **WA School Canteens - packing healthy lunchboxes (59)**  **Weblink:**  <https://www.waschoolcanteens.org.au/schoolcommunity/packing-healthy-lunchboxes/>  **Year developed**:  Unknown  **Last updated**:  2018  **Affiliation:**  WA School Canteen Association Inc.  **Jurisdiction**:  Australia  **Cost:** nil | **Purpose:**  Assist canteens/ other food services to provide & promote healthy choices & operate economically viable & professional businesses  **Target audience:**  Parents of school-aged children  **MARS website quality mean score:** 3.1/5 | 1. Choose items from the 5 food groups to give children the nutrition needed to play & learn for the school day 2. LB rewards & treats don't have to be something edible 3. After school snacks should be just as healthy   **Credibility of information**:   - Messages consistent with ADG & refers to AGHE - No explicit evidence of engagement with health professionals | | **Key Features**:   - General information re. LB & healthy eating - PDF resources – The ABCD of packing healthy lunch boxes (A=B&C, B=F & V, C=reduced fat dairy, meat & alt. & D=snacks), menu planner, top 10 snacks, LB notes & jokes - Links to AGHE and FSANZ (reading food labels)   **Useability**:   - Other pages re. canteens, training, the StarCAP2 & Star Choice program for canteens - Search function   **Social / Community features:** Share content via social media & email, Twitter/FB pages  **Technical features:** Nil | |  |
| **TAS Health - Healthy kids lunchboxes (60)**  **Weblink:**  https://www.dhhs.tas.gov.au/healthykids/early_childhood/lunch_boxes  <https://www.dhhs.tas.gov.au/healthykids/blog/waste_free_lunchboxes>  **Year developed**:  Unknown  **Last updated**:  Unknown  **Affiliation:**  Tas Government, Dept of Health and Human Services  **Jurisdiction**:  Australia  **Cost:** nil | **Purpose:**  Provide families with information about nutrition & PA  **Target audience:**  Parents of school aged children  **MARS website quality mean score:** 3.1/5 | 1. Choose everyday foods from the five food groups 2. Make water main drink 3. Leave out sometimes foods (lollies, chips & chocolates)   **Credibility of information**:   - Messages consistent with ADG - No explicit evidence of engagement with health professionals but likely | | **Key Features**:   - Information re. packing a LB, food safety, waste-free L, tips/ideas - Links to recipes, booklets   **Useability**:   - Part of a larger website with content on a broad range of health topics - Search function   **Social / Community features:** TAS government Move Well Eat Well FB page  **Technical features:** Nil  **Limitations:**   - Navigation difficult, pages not all linked & found in different sections of the website | |  |
| **SA Health - Healthy lunchboxes (61)**  **Weblink:**  <https://www.sahealth.sa.gov.au/wps/wcm/connect/public+content/sa+health+internet/healthy+living/healthy+eating/healthy+eating+tips/healthy+lunchboxes>  **Year developed**:  2012  **Last updated**:  2017  **Affiliation:**  SA Government, SA Health  **Jurisdiction**:  Australia  **Cost:** nil | **Purpose:**  Protect & improve the health of all SA by providing leadership in health reform, public health services, health & medical research, policy development & planning  **Target audience:**  Parents of school aged children, adults  **MARS website quality mean score:** 3.0/5 | 1. Eating a healthy L can help maintain energy levels at work, school or study 2. The best L are the ones you pack at home 3. Packing a healthy L is a great way to boost number of recommended daily serves of the 5 food groups   **Credibility of information**:   - Messages consistent with ADG - No explicit evidence of engagement with health professionals but likely | | **Key Features**:   - Information re. healthy LB (tips, ideas) - Links to internal & external websites including WA Packed with Goodness resource - Six video links from Flinders Uni re. healthy LB   **Useability**:   - Part of a larger website with content on a broad range of health topics - Search function   **Social / Community features:** Share content vis social media / email, Twitter / FB / Youtube / Linkedin account  **Technical features:** Increase/decrease font size  **Limitations:**   - Some broken links | |  |

**Abbreviations**: LB = Lunchbox; F = Fruit; V = Veg; WG = Whole grains; B&C = Breads & Cereals; P = Protein; CHO = Carbohydrate; BF = Breakfast; L = Lunch; D = Dinner; T/A = Take away; PI = Personal Information; FB = Facebook; intro = introduction; alt = alternatives; ADG = Australian Dietary Guidelines; DG = Dietary Guidelines; pref. = preferences; AGHE = Australian Guide to Healthy Eating; FSANZ = Food Standards Australia and New Zealand; SA = South Australians; PA = Physical Activity

Additional 4 -2 :Summary of included apps (Objective three)

| **APP INFORMATION** | | **TOP 3 KEY MESSAGES** | **FUNCTIONALITY** |
| --- | --- | --- | --- |
| **The Ultimate Mix-and-Match School Lunchbox (64)**  **Version 1.2**  **Developer:**  Trellisys.net <https://www.familius.com/52-school-lunches->  **Year developed**: 2013  **Affiliation:** Individual  **Jurisdiction**: USA  **Category^a^**: Lifestyle  **Cost**: Free; Freemium $2.99 | **Platform**: iPhone, iPad and iPod touch  **Purpose:**  Provide parents with L ideas for children  **Target audience:**  Parents & children  **User rating**: Insufficient reviews  **MARS app quality mean score:** 3.4/5 | 1. Include WG, P, F & V at every L 2. Have different combinations of food at L for interest   **Credibility of information**:   - Provides a link to Department of Health in the United States - Authors not trained dietitians/nutritionists - No evidence engagement with health professionals | **Key Features**:   - Provides random meal-combination generator or parents choose own combinations by scrolling through recipes - Recipes are sorted by three food groups (WG, P, F &V) - Freemium upgrade required for access to complete bank of recipes - Shopping list automatically generated, ingredients can be added - Notifications to make a new lunch box - Information section with intro to app, nutrition, healthy eating advice   **Useability**:   - Saves previous lunch boxes for reuse - Bright colours - Easy to navigate   **Data direction:** two-way  **Social / Community features:** ‘like’ features & leave comments  **Technical features:**  Login not required, PI not collected |
| **LaLa Lunchbox**  **Version 3.8.1 (63)**  **Developer:**  Lala Lunchbox, LLC  <http://lalalunchbox.com/>  **Year developed**: 2012  **Affiliation:** Commercial  **Jurisdiction**: USA  **Category^a^**: Food & drink  **Cost**: Free; Freemium $2.99-$5.99 | **Platform**: iPhone, iPod touch, iPad (not optimised)  **Purpose:**  Assist children in choosing LB items whilst giving parents control over food choices available  **Target audience:**  Children supported by parents  **User rating**: Insufficient reviews  **MARS app quality mean score:** 3.3/5 | 1. Include F, V, protein & snack with every L 2. Encourage independence by allowing children to choose their meals   **Credibility of information**:   - No evidence messages based on DG - No evidence engagement with health professionals | **Key Features**:   - Kids design daily LB by choosing from parent-controlled lists of F, V, P & snacks - Shopping list automatically generated - Parents remove/add food items to lists & create personalised items - Add password for parental control - Pay for lists of food groups for specific dietary needs (gluten free) - Notifications to design lunch boxes & buy groceries - Personalise child’s LB & create favourites for individual children   **Useability**:   - Easy layout to follow, minimal clicks needed to edit lunch box - Minimal text on screen - Orders lunch boxes alphabetically - Inadequate colour, significant white space   **Data direction:** mostly user input, minimal information output  **Social / Community features:** Nil  **Technical features:** Login optional, web access not required, PI not collected |
| **Kids Food (62)**  **Version 2.0**  **Developer:** Huyen Trang Nguyen  **Year developed**: 2015  **Affiliation:** Commercial  **Jurisdiction**: Vietnam  **Category^a^**: Food & drink  **Cost**: $5.99 | **Platform**: iPhone, iPod touch and iPad  **Purpose:**  Provide parents with easy meals for children & babies  **Target audience:**  Parents  **User rating**: Insufficient reviews  **MARS app quality mean score:** 2.8/5 | 1. Include variety of foods in child’s LB: fresh F, V, P, dairy & CHO foods 2. Make food healthy by cooking rather than T/A 3. Shopping once a week helps to stay organised   **Credibility of information**:   - No evidence messages based on DG - Recipes not in line with healthy eating guidelines - No references for healthy eating tips - No evidence engagement with health professionals | **Key Features**:   - Child/family recipe categories e.g. kids snacks, kids LB ideas - Search categories & save recipes in favourites - Personalise by saving recipes, creating new recipe groups & adding these to calendar   **Useability**:   - Categories laid out clearly on home page - Navigation around app slightly confusing - Layout of recipes are difficult to read - Photos poor quality   **Data direction:** mostly information output (recipes)  **Social / Community features:** Nil  **Technical features:** Login not required, personal information not collected  **Limitations:** Ads present despite being paid for |
| **Change4Life Smart Recipes**  **Version 3.0.3 (65)**  **Developer:** Public Health England <https://www.nhs.uk/change4life>  **Year developed**: 2013  **Affiliation:** Government  **Jurisdiction**: United Kingdom  **Category^a^**: Food & drink  **Cost**: Free | **Platform**: iPhone, iPod touch and iPad  **Purpose:**  Provide parents & families with healthy recipes, nutrition & healthy eating information & advice on meal planning  **Target audience:**  General & parents  **User rating**: Insufficient reviews  **MARS app quality mean score:** 3.3/5 | 1. Aim for 5 serves F & V per day 2. Choose foods low in added sugars, saturated fat & salt 3. Choose home cooking before bought food; healthier & saves money   **Credibility of information**:   - Information developed from Public Health England’s DG (‘The Eatwell Guide’) - Developed by Government Department of Health (doesn’t state which health professionals were consulted) | **Key Features**:   - Provides family friendly recipes for BF, L, D, desserts & snacks - ‘Meal-mixer’ randomly generates 3 recipes for the day (BF, L, D) - Recipes provide nutrition information & facts - Shopping list automatically generated - Includes healthy eating advice, seasonal tips, cooking advice & cooking terminology - Personalise by making favourites list, editing shopping list & selecting dietary requirements   **Useability**:   - Easy to navigate, video instructions easy to follow   **Data direction:** mostly information output (recipes, nutrition information)  **Social / Community features:** Share to social media groups or join Change4Life community for additional support  **Technical features:** Not password protected, notifications, PI not collected  **Limitations:** One broken link to Change4Life Food Scanner |

^a^AppStore Category;
**Abbreviations**: LB = Lunchbox; F = Fruit; V = Veg; WG = Whole grains; P = Protein; CHO = Carbohydrate; BF = Breakfast; L = Lunch; D = Dinner; T/A = Take away; PI = Personal Information; FB = Facebook; intro = introduction; DG = Dietary Guidelines
